# Unleashing the Potential of Electroactive Hybrid Biomaterials and Self-Powered Systems for Bone Therapeutics

**DOI:** 10.1007/s40820-024-01536-9

**Published:** 2024-10-17

**Authors:** Shichang Liu, Farid Manshaii, Jinmiao Chen, Xinfei Wang, Shaolei Wang, Junyi Yin, Ming Yang, Xuxu Chen, Xinhua Yin, Yunlei Zhou

**Affiliations:** 1 Honghui Hospital, Xi’an Jiaotong University, Xi’an, 710018 People’s Republic of China; 2 Department of Bioengineering, Henry Samueli School of Engineering and Applied Science, University of California Los Angeles, Los Angeles, 90095 USA; 3 Hangzhou Institute of Technology, Xidian University, Hangzhou, 311231 People’s Republic of China

**Keywords:** Electroactive biomaterials, Self-powered bioelectronics, Bone regeneration, Bone tissue

## Abstract

The incidence of large bone defects caused by traumatic injury is increasing worldwide, and the tissue regeneration process requires a long recovery time due to limited self-healing capability. Endogenous bioelectrical phenomena have been well recognized as critical biophysical factors in bone remodeling and regeneration. Inspired by bioelectricity, electrical stimulation has been widely considered an external intervention to induce the osteogenic lineage of cells and enhance the synthesis of the extracellular matrix, thereby accelerating bone regeneration. With ongoing advances in biomaterials and energy-harvesting techniques, electroactive biomaterials and self-powered systems have been considered biomimetic approaches to ensure functional recovery by recapitulating the natural electrophysiological microenvironment of healthy bone tissue. In this review, we first introduce the role of bioelectricity and the endogenous electric field in bone tissue and summarize different techniques to electrically stimulate cells and tissue. Next, we highlight the latest progress in exploring electroactive hybrid biomaterials as well as self-powered systems such as triboelectric and piezoelectric-based nanogenerators and photovoltaic cell-based devices and their implementation in bone tissue engineering. Finally, we emphasize the significance of simulating the target tissue’s electrophysiological microenvironment and propose the opportunities and challenges faced by electroactive hybrid biomaterials and self-powered bioelectronics for bone repair strategies.

## Highlights

- Introduce the role of bioelectricity and the endogenous electric field in bone tissue and summarize different techniques to electrically stimulate cells and tissue.
- Highlight the latest progress in exploring electroactive hybrid biomaterials as well as self-powered systems such as triboelectric and piezoelectric-based nanogenerators and photovoltaic cell-based devices in bone tissue engineering.
- Emphasize the significance of simulating the target tissue’s electrophysiological microenvironment and propose the opportunities and challenges faced by electroactive hybrid biomaterials and self-powered bioelectronics.

## Graphical abstract

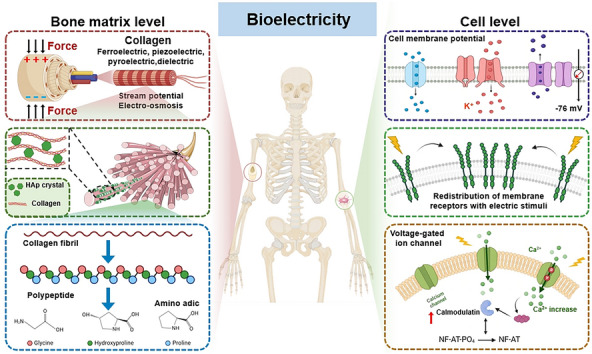

## Introduction

Bone diseases, ranging from arthritis and osteoporosis to bone cancer and fractures, represent significant challenges and burdens in modern society [1–4]. The increasing incidence of large bone defects caused by traumatic injuries, coupled with the limited self-healing capability of bone tissue, necessitates the development of advanced therapeutic strategies [5]. Traditional methods for bone repair, such as autografts and allografts, often come with limitations, including donor site morbidity, limited availability, and risk of immune rejection [6]. Consequently, there is a pressing need for innovative approaches that can enhance bone regeneration and repair.

One promising avenue in bone therapeutics is the integration of electroactive hybrid biomaterials (EHBs) and self-powered systems [7, 8]. These technologies leverage the intrinsic bioelectrical properties of bone tissue to create biomimetic environments that promote bone healing. The concept of bioelectricity in bone tissue is not new [9]; it has long been recognized that electrical signals play a crucial role in bone remodeling and regeneration. Natural bone is a composite material primarily composed of hydroxyapatite and collagen, which exhibits specific bioelectric phenomena under different physiological conditions. These phenomena include dielectric properties, pyroelectricity, ferroelectricity, and piezoelectricity in dry bone [10, 11], as well as stream potential and electroosmosis in wet bone [12–14].

Given the critical role of bioelectricity in bone tissue, researchers have explored the use of exogenous electric fields (Exo-EFs) to mimic or modulate Endo-EFs for treating bone diseases. Exogenous electrical stimulation (ES) has been widely considered an external intervention to induce the osteogenic lineage of cells and enhance the synthesis of the extracellular matrix, thereby accelerating bone regeneration. The US Food and Drug Administration has approved the use of ES as a non-pharmacological treatment for fracture healing, with devices available in both invasive and noninvasive forms. The development of EHBs that simulate the electrophysiological microenvironment of bone tissue has attracted significant attention. EHBs combine the properties of electroactive materials with biocompatibility and biodegradability, making them suitable for bone tissue engineering. Conductive biomaterials (CBMs), such as metallic CBMs and metal nanoparticles, facilitate the regulation of cell–cell or cell-extracellular matrix (ECM) cross talk by enhancing electron transport at the interface between bone cells or tissue and materials. Piezoelectric biomaterials, including piezoceramics and piezopolymers, can induce the electrophysiological environment required for bone regeneration by surface polarization.

The application of EHBs in bone therapeutics involves promoting osteogenesis, suppressing osteoclast activity, immunomodulation, vascularization enhancement, antibacterial effects, and drug delivery. EHBs can serve as delivery platforms for therapeutic drugs or sensors, assisting in treatment and monitoring prognosis. Self-powered bioelectronic devices, such as triboelectric nanogenerators (TENGs) and piezoelectric nanogenerators (PENGs), offer non-pharmacological rehabilitation methods that enable bioelectronic therapies beyond what is possible with pharmaceuticals. These devices can regulate the activity of excitable cells or bone tissue, promoting bone regeneration and accelerating functional recovery. The integration of electroactive hybrid biomaterials and self-powered systems into bone therapeutics holds transformative potential. These technologies offer innovative solutions for enhancing bone regeneration and repair, addressing the limitations of traditional methods. As research continues to advance, the convergence of bioengineering, materials science, and nanotechnology is expected to revolutionize the field of bone therapeutics, ultimately improving patient outcomes and quality of life [15–24].

This review focuses on summarizing the current state of the art in electroactive biomaterials and self-powered electrical stimulation devices that replicate the electrophysiological microenvironment for bone tissue regeneration. It highlights conductive and piezoelectric biomaterials as well as passive self-powered electrical stimulation technologies, primarily TENG, PENG, and photovoltaic cells. First, we discuss the nature and mechanism of the formation of endogenous bioelectricity in bone tissue. Second, we summarize and discuss electroactive biomaterials with different structures and properties, with a particular emphasis on their applications in treating bone-related diseases. Finally, we provide an overview of the current applications of electroactive biomaterials and self-powered devices in the field of bone tissue engineering and propose potential obstacles that may be encountered in future clinical translation.

## Bone Bioelectricity and Cell-Bone Interaction with Electrical Stimulation Signals

The discovery of Endo-EFs can be traced back to the eighteenth century when Luigi Galvani made a breakthrough in studying the contraction behavior of frog sciatic nerve muscles, pioneering new research avenues for the interplay between electricity and biology [25, 26]. Currently, Endo-EFs phenomena are defined as the electrical potential and polarity changes occurring in organs, tissues, and cells during biological activities such as water molecules, ions (Ca^2+^, Na^+^, K^+^, Cl^−^, PO_4_^2−^), and transmembrane potential [27, 28]. As critical indicators reflecting cell morphogenesis and tissue function formation, bioelectricity signals are influenced by ion channel proteins, ion pumps in the cell membrane, and electrical synapses or gap junctions [29]. These signals participate in regulating various biological processes, including cell migration, proliferation, differentiation, nerve conduction, muscle contraction, embryonic development, and tissue regeneration [30]. Consequently, investigating Endo-EFs significantly contributes to understanding the physiological and pathological processes of living organisms. Simulating and reshaping the electrical microenvironment can activate the electrophysiological behavior of injured cells or disabled tissues, thereby realizing disease treatment.

Endogenous piezoelectricity is another significant electrical property in bioelectricity. As the name implies, piezoelectricity describes the ability of materials to generate electricity in response to applied mechanical deformation, including tension, bending, compression, vibration, and so on [31, 32]. When piezoelectric materials are subjected to a specific direction of external force (such as an ultrasonic wave), they deform, causing internal polarization and the relative surface to produce positive and negative opposite charges. When the external force is removed, the piezoelectric material returns to its uncharged state, which is known as the positive piezoelectric effect. The electric field will deform the piezoelectric material along its polarization direction. When the electric field is removed, the deformation of the piezoelectric material disappears, known as the inverse piezoelectric effect [33]. In the physiological environment, the piezoelectric response originates from the anisotropic structure of biological components or transient deformations, contributing to the generation of nonzero electric dipole moments [34, 35], which are widely regarded as closely related to bone formation and remodeling. Extensive research has confirmed the presence of piezoelectricity in many biological macromolecules, tissues, and organs, including amino acids, proteins, DNA, bone, cartilage, ligaments, tendons, and hair [33, 36, 37]. At the molecular level, amino acids exhibit structure-dependent piezoelectric properties. This is attributed to the chiral symmetric groups present in most amino acids, thus giving rise to inherent polarity along the molecular chain formed by left- or right-hand structures. Additionally, piezoelectricity within peptides is primarily induced by hydrogen bonds oriented along helical axes. Once subjected to transient stress from the external environment, the displacement of hydrogen bonds leads to the rearrangement of dipole moments on the molecular chain, causing polarization and generating accumulated charges. Collectively, the structural features of amino acids and peptides lay the foundation for the piezoelectric properties of proteins. Although the piezoelectric coefficients of these natural piezoelectric biomacromolecules range from 0.01 to 10 pm V^−1^, they are crucial for maintaining the electrical properties of tissues.

**Fig. 1:**
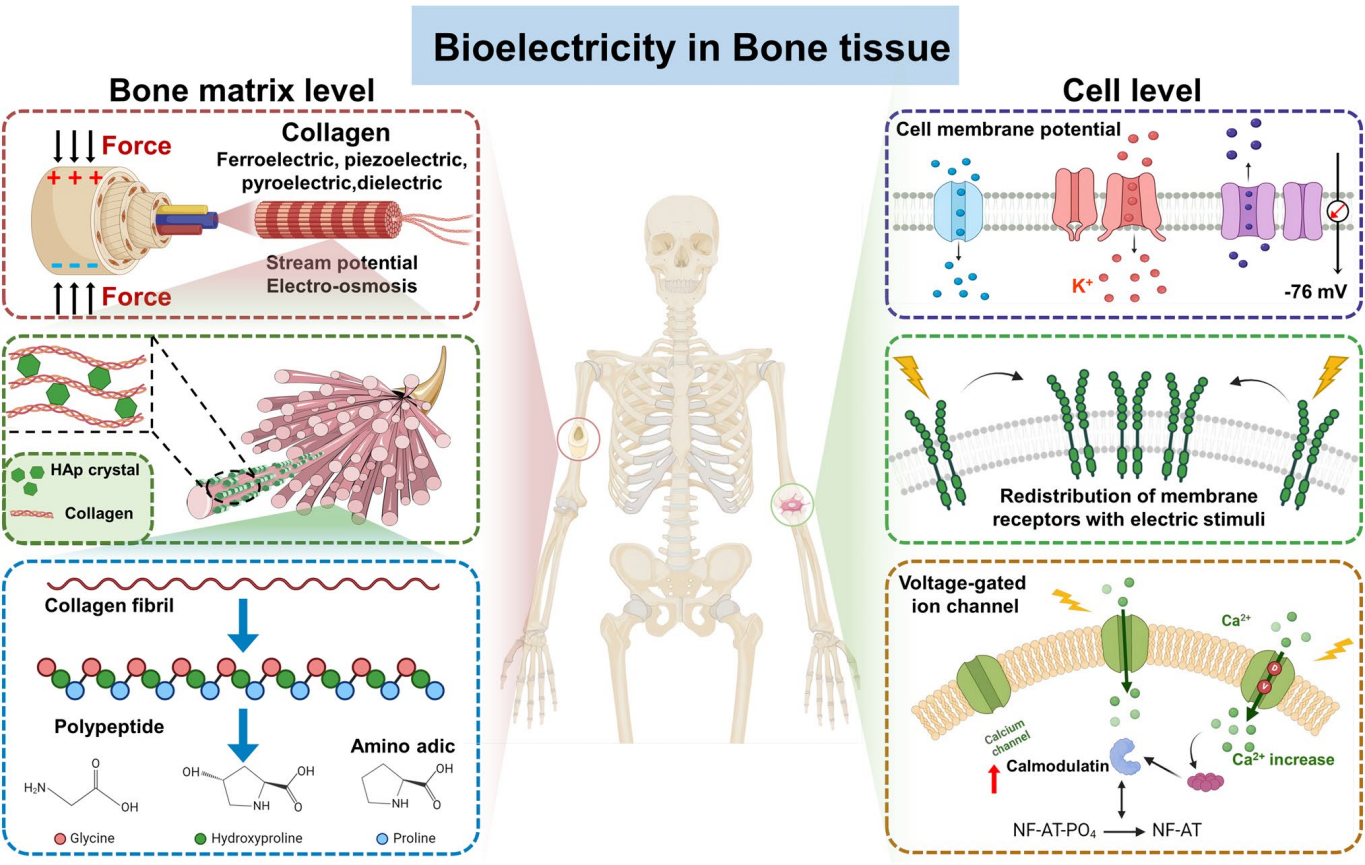
Schematic diagram of the endogenous bioelectricity in bone tissue

Natural bone is generally regarded as a composite material in which hydroxyapatite mainly forms the inorganic portion (69 wt%) with a collagen matrix (22 wt%) embedded in it, showing a densely packed fibrillar state [27]. Due to the physiochemical properties and anisotropic microstructure of these components, bone tissue encompasses specific bioelectricity phenomena under different physiological conditions, which combine dielectric properties, pyroelectricity, ferroelectricity, and piezoelectricity for dry bone, along with stream potential and electroosmosis for wet bone (Fig. 1). Investigations have uncovered the valuable effects of electrical stimulation generated by intrinsic electric fields or exogenous electrical signals through applying electrically conducting materials or devices on bone restoration or the management of bone-related diseases, including osteoporosis, bone fractures, and bone tumors [2, 38].

In terms of components, the bioelectrical characteristics of bone tissue are mainly attributed to collagen and hydroxyapatite. It has been demonstrated that the dielectric properties arise from the separation of hydrogen bonds between collagen and hydroxyapatite by applying external electrical fields [39]. The dielectric constant of bone is likely contingent upon the water content and electric frequency, which can be quantified using impedance spectroscopy techniques [40, 41]. Phadke et al. reported that the dielectric constant of bone decreased with elevated frequency [42]. Also, mineralized bone experiences a reduction in dielectric constant and conductivity compared to pure dry bone, which might be attributed to the breaking and disassembly of hydrogen bonds as well as the reduction of the freedom of side chains inside the phase of mineralized collagen [43]. Recent investigations revealed a significant interrelation with bone mineral density (BMD) [44], suggesting that the dielectric constant can serve as a feasible indicator to evaluate the mechanical performance and health status of bone or even for the diagnosis of osteoporosis. Pyroelectricity can be directly connected to the peculiar structure of the folding helix amino acid residue chains in collagen fibrils. Temperature variation disturbs the symmetry of the triple helical nature within the collagen molecule, resulting in a nonlinear polarization response, thus generating thermoelectric potential [45]. Furthermore, collagen fibers can change the orientation of polar groups and exhibit diverse alignment within bone plates, similar to the alignment of ferroelectric domains. The existence of residual polarization and permanent dipoles imparts the collagen component in bone with a ferroelectric nature [46].

Early in 1957, the piezoelectric property in bone was proven by Fukada, who initially quantified the value of the piezoelectric constant as one-tenth that of quartz [10]. Current scholarship has figured out that the origin of the piezoelectric effect comes from the non-centrosymmetric conformation of collagen fibrils sliding against each other [47, 48]. As the fundamental protein of bone tissue, collagen exhibits self-assembly into a triple helix structure through hydrogen bonds between -NH_2_ and -C = O-, sequentially stacking to form a quasi-hexagonal lattice crystalline. When subjected to mechanical stress, the helical chains undergo bending and relaxation deformation due to hydrogen bonds translocation, resulting in a change in axial polarization, thus leading to the piezoelectric effect. Researchers have also detected piezoelectric responses in hydroxyapatite blocks, supposing that hydroxyapatite might synergistically form piezoelectric bone tissue with collagen [49]. However, as the major inorganic component, the high rigidity of HAp dampens the mechanical perception of collagen fibrils to external force and restricts the entry and flow of water molecules to form polar hydrogen bonds, thus attenuating the generation of piezoelectric stimulation [50]. Meanwhile, intrinsic piezoelectric heterogeneity exists within single collagen fibrils, which even confers anisotropic piezoelectricity to bone tissue [51]. This means that the piezoelectric properties vary across different bone regions. For example, the femur presents a piezoelectric coefficient of 0.7 pC N^−1^ under shear stress, whereas the tibia displays a higher value ranging from 7.66 to 8.48 pC N^−1^ [52]. As explained by Wolff’s law, bone can respond to mechanical loading due to the induced polarization by the piezoelectric effect, thus facilitating bone remodeling and repairing [53]. Different from dry bone samples, investigations suggested that the electric potential in wet bone is induced by stress-triggered ion flows in the bone interstitial fluid, called streaming potential, rather than the piezoelectric effect [54]. As the presence of water molecules increases the conductivity of the bone matrix, it weakens the piezoelectric characteristics of collagen [55]. Owing to the endogenous electric field, the calcium fluxes appearing in the interstitial fluid through the canaliculi and lacunae are governed by the electroosmosis phenomenon [52].

Collectively, the electrophysiological characteristics of bone tissue are an overlap and collective of dielectric properties, pyroelectricity, ferroelectricity, piezoelectricity, stream potential, and electroosmosis. Once bone trauma emerges, the relatively static electric potential will encounter damage at the injury site. Taking bone fracture as an example, the injured tissue, including distal bone fragments and the traumatized site, displays noticeable negative potential, which slowly reverts to a normal state as the healing progresses [56]. Hence, many researchers have proposed that the remodeling of the electrophysiological microenvironment may lay the foundation for the application of Exo-EFs in an invasive or noninvasive manner. With ongoing progress and increasingly in-depth exploration of the formation mechanism and biological functions, the efficacy of endogenous bioelectricity provides novel feasible paradigms and targets for bone-related disease therapy. Consequently, the exertion of material design to assist in building up Exo-EFs to mimic or modulate Endo-EFs for bone disease treatment has emerged as a cutting-edge focus in the field of biomaterials and regenerative medicine [57, 58].

## Ways of Delivering Electrical Signals to Bone Tissue

### Exogenous Electrostimulation Source

Inspired by endogenous bioelectricity, electrostimulation, simulating the Endo-EFs to modulate the bioelectric state and accelerate fracture healing, comes to the fore as a promising non-pharmacological treatment in clinical practice and has been approved by the US Food and Drug Administration. The clinic-available ES devices mainly include invasive and noninvasive aspects, and the electric stimuli are generally induced by using direct current (DC) or alternating current (AC) [59, 60]. Given that the EXO-EFs are easily affected by the tissue environment, adjusting the ES parameters as needed is beneficial for constructing protocols that can positively regulate cell behaviors and accelerate the improvement of in vitro research on the formation and mechanism of endogenous bioelectricity.

DC is regarded as the common and most feasible method widely used for most in vitro assays by directly inserting electrodes into the culture medium [61]. By using the connected commercial electric stimulator, the amplitude of voltage/current and duration can be conveniently altered [62]. DC stimulation is one of the most efficient methods of ES therapy. It is invasive for bone defects by implanting the cathode in the fracture area, while the anode is fixed in adjacent soft tissue. It was found that DC stimulation (100 mV mm^−1^, 1 h day^−1^) can effectively promote osteogenesis in the presence of osteogenic-induced factors [63], while 200 mV mm^−1^ enhanced early stem cell differentiation and bone matrix production without additional chemical inductive factors [64]. In another report, both high DC EFs (10–15 V cm^−1^) and low DC EFs (smaller than 5 V cm^−1^) were able to elevate intracellular calcium levels, promote cell elongation, and guide the motility of osteoblast-like cells [65]. Further development of DC-based ES therapy is largely limited by its disadvantages, such as thermal damage, formation of capacitive bilayers, toxicity of metal-based electrodes, pH alteration, and by-products from electrolysis [66]. In AC electrical stimuli, besides the level of voltage/current and stimuli duration, the frequency (Hz), pulse width (sec), periodical time, and especially the type of wave (monophasic, biphasic, sine, square, triangular, etc.) can also be controlled [67]. Bader et al. used sinusoidal AC with square voltage (0.1, 1.4, and 2.8 V) at a frequency of 20 Hz to evaluate its effect on human osteoblasts. Results suggested that higher electrical stimulation with 2.8 V significantly reduced osteogenic differentiation, while a suitable voltage amplitude (0.2 and 1.4 V) did help osteogenic gene expression [68]. Additionally, AC stimulations can be applied in a noninvasive mode. In early work exploring how fixed ES affected osteoblast-like cells’ behavior and bone formation on the PCL-based 3D-printing scaffolds, to minimize cell damage, the plate electrodes were separated with an insulator containing culture medium and cell-loaded scaffolds. An AC electric field of 55 ± 8 mV cm^−1^ at a frequency of 60 Hz was exerted between a pair of electrodes to produce a sinusoidal signal [60].

Up to now, there exist several pathways to impose ES on target cells or tissues, including direct stimulation, capacitive stimulation, inductive stimulation, and combined stimulation [69, 70]. Commercial electrical stimulators are commonly used for direct electrostimulation, especially for in vitro experiments in the laboratory [71]. The relative electrical parameters can be conveniently and quickly tuned, helping to precisely screen out the optimal parameters and develop a standard protocol for regulating cells or bone tissue. The human body’s response to current and tolerance varies; the average human body may feel the stimulation of a current value of about 1 mA, while the human body experiences muscular contraction pumping at 5–20 mA. It is worth noting that when the current of electrical stimulation exceeds 20 mA, it may cause direct cell death by instantly burning cells and destroying cell membrane structure [72]. Therefore, the biological safety of direct electrical stimulation needs further evaluation.

Inductive coupling (IC) stimulation refers to generating an induced electric field through a conductive coil surrounding the cell culture platform [73]. Subsequently, the alternating current generated flows through the coil, producing a magnetic field and an alternating electric field perpendicular to the magnetic field direction. Compared to simple direct electrical stimulation, IC effectively avoids direct contact between cells and electrodes, thereby eliminating the presence of toxic by-products [74]. A recent review analyzed 117 studies using IC-ES to treat bone and cartilage (73 in animal models, 44 in human patients). It was found that in animal studies, a pulse frequency > 7.5 Hz and in human patients > 15 Hz resulted in better efficacy. Additionally, in preclinical and clinical studies, magnetic field MF intensity > 1.0–1.2 mT was associated with higher healing rates. However, clinical evidence is still scarce for each condition tested, particularly for implants’ osseointegration, osteoporosis, and osteoarthritis in humans [75]. Capacitive coupling (CC) is also a noninvasive electrical stimulation method. By placing the cell culture system between two parallel capacitor plates connected to a generator, the generated electric field is evenly transmitted through the culture medium to the cells regardless of their position in the cell culture well [76]. The entire system often does not directly contact the cells. This allows for uniform stimulation of cells through the cell culture medium regardless of their position in the cell culture well. However, the development of CC-ES is still severely hindered by many objective factors, such as complicated circuit design, weak anti-interference capabilities, and inevitable attenuation in bone tissue, making it difficult to further develop and study [73, 76].

For a long time, exogenous electrical stimulation has been directly applied to cell culture mediums or local tissues, including deep brain stimulation, activation of neurons and CMs, modulation of stem cell differentiation, and tissue repair activation. However, these flat electrodes have shown many limitations in practical applications. Firstly, the macroscopic electric field generated lacks selectivity and spatial resolution, causing severe side effects on non-targeted cells or tissues. Secondly, due to the absorption effects of the liquid environment and adipose tissue, the cell culture medium or surrounding tissue weakens the voltage threshold applied to the target cells/tissues. Conductive biomaterials with micro/nanostructures can serve as new electrode materials, providing additional electrical stimulation not exceeding safe thresholds based on physiological needs, and can be used as a bio-scaffold in direct contact with cells or tissues along with piezoelectric biomaterials, thus avoiding the influence of surrounding media. Additionally, through the rational design of morphology, physicochemical properties, and mechanical properties, conductive biomaterials can synergistically guide cell adhesion, proliferation, and differentiation. Therefore, electrical stimulation based on conductive biomaterials can provide a diverse platform for regulating cell or tissue behavior and intervening in tissue repair.

### Self-Powered Electrostimulation Source

External electric stimulators and implantable medical electronics (IMEs) used in clinics are generally powered by batteries. The constant battery capacity greatly limits the device’s lifespan. Regular replacement of power sources not only pollutes the environment but also increases the economic and mental burden on patients. In addition, batteries account for most of the weight and volume of IMEs. In recent years, scientists have proposed converting human kinetic energy, thermal energy, or chemical energy in the physiological environment into electricity for self-powered IMEs, enabling the devices to operate normally without an additional power supply. Except for ambient light and temperature, in vivo redox reactions and endocochlear potential, there is a significant amount of untapped small biomechanical energy in the human body, such as muscle contractions, vocal cord vibrations, lung respiratory movements, heartbeats, and blood flow. Therefore, various kinds of self-powered ES devices have been developed, including PENGs [77], TENGs [78], photovoltaic cells (PVCs), automatic wristwatches (AWs) [79], pyroelectric nanogenerators (PYENGs) [80], biofuel cells (BFCs) [81], and endocochlear potential (EP) collectors [82]. Considering the special microenvironment of bone tissue, this section will focus on reviewing TENG, PENG, and PVC as self-powered ES sources for bone tissue engineering.

#### TENG

**Fig. 2:**
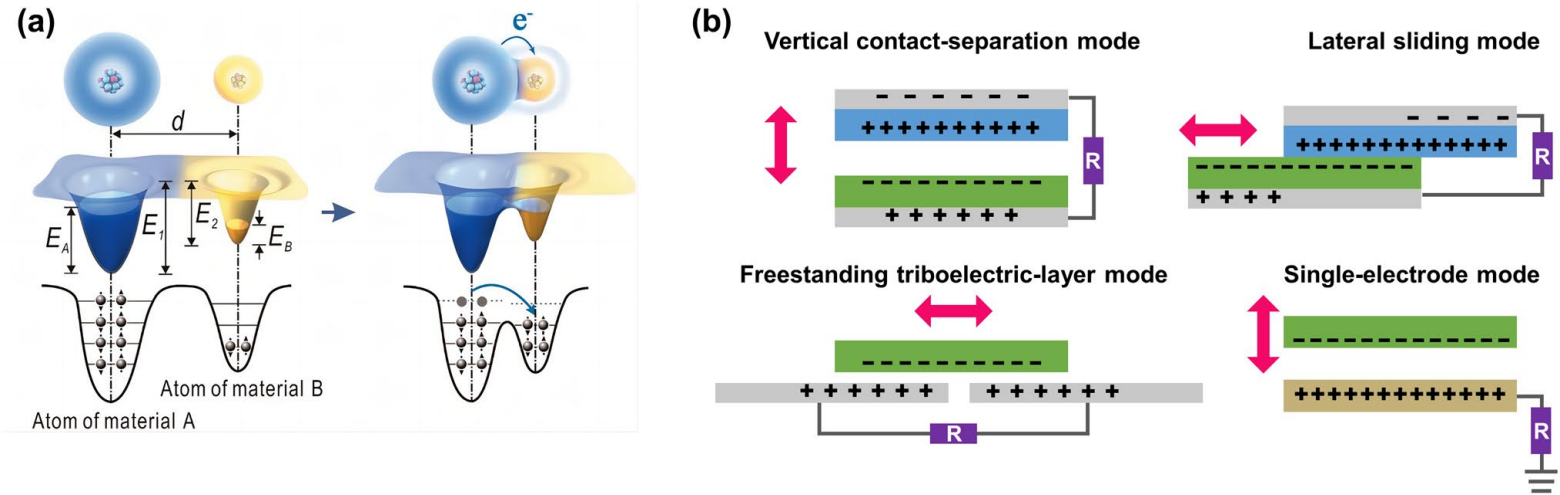
Working principle of TENGs. **a** Electron-cloud-potential-wall model. Reproduced with permission [85]. Copyright 2018, Wiley–VCH. **b** Four fundamental modes of TENG. The theoretical model of TENGs is based on the displacement current of Maxwell’s equations [86]

TENGs were first proposed by Wang’s group early in 2012 and have been considered a promising type of self-powered device within the field of biosensors and self-powered biomedical devices [83, 84]. The principle of TENGs was inspired by the natural phenomenon of electrification by friction and is described as the combined effect of electrostatic induction and triboelectrification. The basic working mechanism is as follows (Fig. 2a): When two dissimilar materials with opposite electronegativity contact each other upon external force, electric charges will transfer from one to the other material with stronger electron-accepting capability. Once the surfaces of the two materials are separated, large quantities of equal and opposite charges accumulate, creating a potential difference between the electrodes and electrons flowing in the external circuit to form a current [78, 85].

According to the structure and operating modes, TENGs can be categorized into four types, adapting to various application scenarios: vertical contact-separation, lateral sliding mode, single-electrode, and freestanding triboelectric-layer mode (Fig. 2b) [87]. Li’s group and Cao’s group both used the same triboelectric materials, PTFE and Al, to develop wearable TENGs in a contact-separation and sliding friction mode, respectively [88, 89]. TENG-induced ES promoted the differentiation of osteoblasts and also rejuvenated aged BMSCs, regaining their proliferative capacity, pluripotency, and osteogenesis potential. The invention of TENG technology has greatly addressed the power supply issue of traditional electrical stimulators. Recently, the application of TENGs as biomedical sensors has made a significant impact, as they can real-time, self-powered, and portable convert biomedical signals into measurable electric signals [90, 91]. This serves the early diagnosis and prognosis monitoring of diseases, enabling individualized and precise health management for patients. Because of the wet and hostile interior environment of the human body, the implantable TENG requires protection from external forces, as its operating mechanism relies on the complete absence of water and moisture conditions. To that purpose, TENG encapsulating layers made of biocompatible materials have been developed to tolerate implanted devices for an extended period. These packaging layers marginally limit the TENG’s power output. Furthermore, researchers must constantly explore and experiment to find the electrical stimulation that has the most effect on cell activity while causing the least harm to the body. Although TENG-ES has numerous beneficial impacts on bone and wound healing processes, there is limited direct data to explain how this ES stimulates osteoblast and fibroblast development. More research should be done to directly verify the causal association between osteoblast and fibroblast differentiation and ES.

In the field of bone tissue regeneration research, there are increasingly more reports on TENG-related applications, primarily focused on determining cell osteogenesis, accelerating bone healing [92], sensing for disease-monitoring [93], mitigating bone aging [94], and inhibiting implant-related infections [95]. By designing in wearable or implantable modes, TENGs can directly serve as a self-powered energy source, providing direct electrical stimulation and participating in physiological activities on or within the human body, thereby paving an alternative way for modern medical treatment and intervention. Due to the high electrical output and mechano-sensitivity with cheerfully available and affordable materials, TENGs are expected to provide feasible ES therapy for clinical orthopedic treatment as well as sensing of the treatment process and sustainable power supply for medical devices. Given the portability, patients are likely to be freed from the burden of heavy medical facilities, thereby reducing their therapy and time costs, and creating new paradigms for personalized health management and precise treatment.

#### PENG

**Fig. 3:**
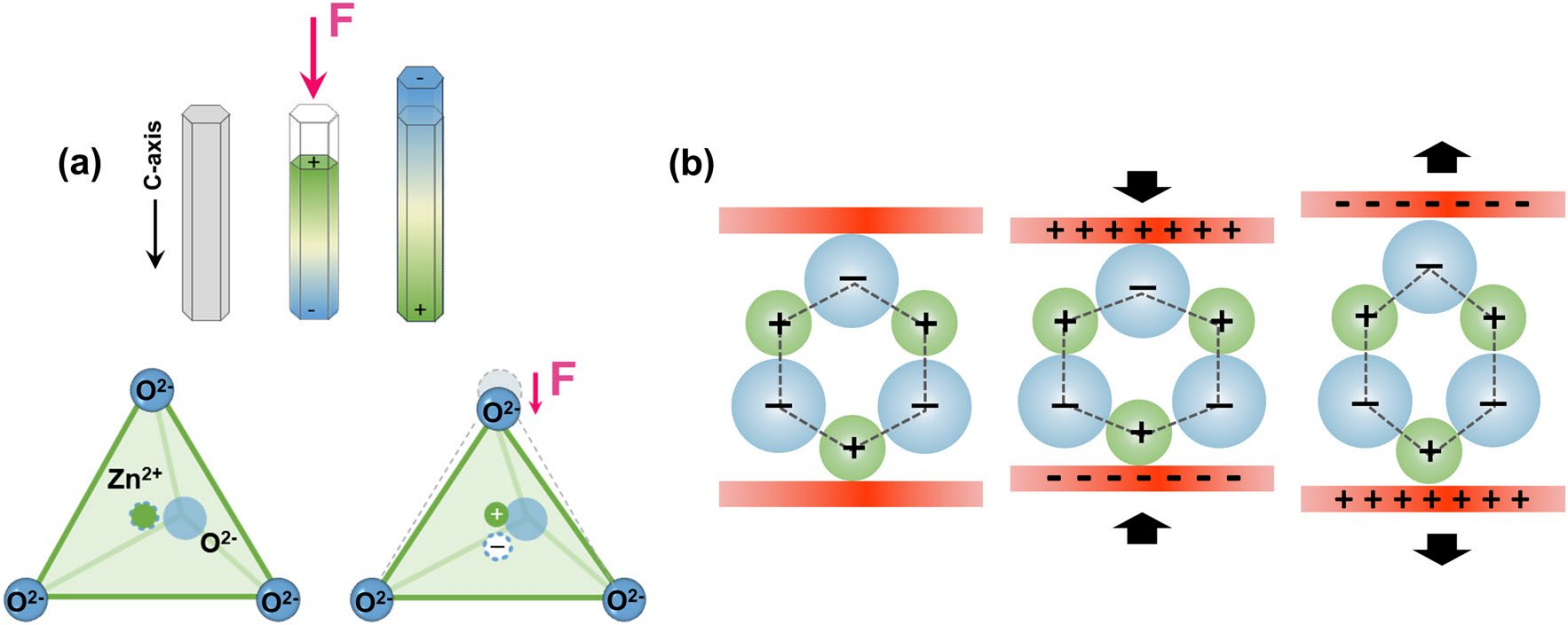
Working mechanism of PENGs. **a** Crystalline structure of ZnO and numerical piezopotential distribution along a single ZnO nanowire under axial stretching or compression. **b** Piezopotential of PENG in compression and tension modes

PENGs, based on the piezoelectric effect, convert external mechanical energy into electrical energy through its deformation and deliver it to the electrode or power electronic devices. The first study about PENG-based self-powered devices can be traced back to 2006 [96] when Wang and Song developed an aligned zinc oxide (ZnO) nanowire array-based PENG to harvest vibration energy and power nanodevices. In the presence of load, the output voltage was about 8 mV [96]. Further, in 2010, Li et al. proposed a ZnO-based single-wire PENG, which generated an alternating current of about 1 and 30 pA when implanted on the diaphragm and heart surface, respectively. This work represents the first successful attempt to apply PENGs to collect and convert in vivo biomechanical energy into electricity [97]. Here, we take ZnO crystals as an example to illustrate the fundamental mechanism of PENGs. As a typical piezoceramic, ZnO possesses a hexagonal structure with non-central symmetry in the direction of the c-axis. As shown in Fig. 3a, Zn^2+^ cations and neighboring O^2−^ anions are tetrahedrally coordinated, and the charge centers of Zn^2+^ and O^2−^ overlap with each other when no external stress is applied; therefore, no polarization is produced. Once stress is exerted along its *c*-axis, the charge centers of cations and anions are relatively displaced, generating a dipole movement in the unit cell [31, 98]. Subsequently, a potential distributed along the stress direction is macroscopically induced after dipole moments’ superposition occurs inside the crystals (Fig. 3b). This is called piezoelectric potential (piezopotential), which can drive the flow of electrons in an external load circuit when the material undergoes mechanical deformation.

The currently available PENGs are mostly composed of external loads, piezoelectric components, and flexible substrates. Its capability to respond to mechanical force and directly generate electrical signals empowers the PENG with outstanding advantages in the application of energy harvesting, biosensing, and therapeutic electrical stimulation [99–101]. Since the performance of a single PENG is mainly determined by the intrinsic piezoelectricity of the piezoelectric components, it is crucial to select the materials and precisely design the device structure for better outcomes. Limited by the finite mechanical deformation pathways and weak deformation amplitudes in tissue engineering, there are few reports of PENG devices being used in orthopedics. Meanwhile, the piezoelectric materials used for bone tissue can be generally divided into three types: flexible artificial piezopolymers like poly(L-lactic acid) (PLLA), polyvinylidene fluoride (PVDF) and its copolymers, natural piezopolymers like collagen, cellulose, and piezoelectric composites by integrating inorganic piezoelectric particles into piezopolymer substrates.

#### TENG-PENG

Both TENG and PENG can convert mechanical energy (heartbeat, pulse, respiration, blood flow, joint motion, etc.) into electricity, which can be harvested and used for direct electrical stimulation or powering implanted medical devices, each having its advantages and drawbacks. PENGs endure long-term bending with good stability but are limited by low output performance. TENGs possess relatively higher voltage output but exhibit low frictional efficiency and require necessary encapsulation to protect the triboelectric layer from erosion and dysfunction in the body’s liquid environment. Particularly for implantable devices, the sensitivity and energy conversion efficiency of NGs need to be quite high due to the subtle magnitude of movement inside the animal body. Currently, exploration and research in this field are mainly focused on enhancing energy conversion efficiency, optimizing device structure, and developing novel friction materials.

By integrating PENG into TENG, it has been proven that the formed TENG/PENG hybrid coupling nanogenerator can not only utilize the advantages of multiple energy conversion mechanisms but also enhance energy conversion efficiency and achieve complete self-powered property. A modified electron-cloud potential-well acceleration theory will account for the enhancement of energy conversion efficiency from a microscopic level [85]. In the intrinsic theory of TENG, the charge transfer is affected by the relative capability of gaining or losing electrons, called surface potential difference, which intrinsically depends on the pairing materials themselves. When a piezoelectric field is introduced by PENG, piezoelectric potential remarkably elevates the potential energy of the electron-cloud potential well, causing friction charges to be bound in the polarization direction due to the difficulty of orbital transition, resulting in accelerated electron transfer from the positive to negative materials [102].

#### Photovoltaic Cell

Besides mechanical energy, in vivo glucose redox energy and chemical energy, ambient temperature, humidity, and light can all be self-powered sources to generate electricity in a battery-free manner. Among them, optical illumination is regarded as a spatiotemporally controllable, wireless, and noninvasive means to modulate material responses. Organic photovoltaic cells (OPVs) converting light into controllable electric current have gained much attention in the field of wireless self-powered health diagnostics and therapeutics. Additionally, the compatibility of OPV cells guarantees biosafety for use in direct contact with cells, tissues, and organs. Traditional silicon-based neuromorphic sensors are a viable option, but they have inherent constraints that are difficult to overcome. First, they have low biocompatibility, which means they may be rejected by biological systems or cause negative reactions when they come into touch with them. Second, the circuitry of these devices is frequently complicated, increasing the difficulties of manufacture and maintenance while simultaneously reducing energy efficiency. Finally, silicon-based devices operate fundamentally differently from ion signal modulation in biology, making it challenging for them to mimic biological systems. Because real neurons interact with one another by regulating flux and polarization through ion species, and organic semiconductors can combine ion polarization with charge transport regulation, organic materials are an ideal candidate for building biomimetic electronics. Organic semiconductor materials are a promising alternative to silicon-based photodiodes of photovoltaic cells, as they offer higher efficiency, pleasant flexibility, lighter weight, lower cost, and simpler production. The charge carriers generated by the photoelectric effect can increase the conductivity of bare silicon linearly with the intensity of light. The introduction of p-n junctions is beneficial for creating controlled PVCs with high spatial selectivity. Jaume et al. reported a silicon-based photovoltaic microcell array (PVMA) platform to stimulate osteoblast-like Saos-2 cells [103]. Through advanced photolithography and e-beam evaporation, the PVMA array had a rectangular morphology of 5 μm × 7 μm and produced photocurrents of 56.53 and 72.9 nA under white LED light and solar illumination, respectively. Results showed that wireless ES triggered intracellular Ca^2+^ activity in 46% of Saos-2 cells. Similarly, the Schottky junction formed at the surface of silicon can also assist in improving light-induced surface charge accumulation. Lin’s group proposed light-responsive graphene-transferred silicon (Gr-Si) with a Schottky junction to promote the osteogenic differentiation of BMSCs with the illumination of a blue LED flashlight at a wavelength of 450 nm [104]. The interface within Gr-Si regulated the surface potential of materials by affecting the positive/negative charge accumulation, which interrupted the expression of upstream signaling molecules. However, the biggest challenge for PVC applied in bone regeneration is tissue penetration. Most photoelectric devices are activated by visible or ultraviolet light, whose wavelengths are easily absorbed by the epidermis and fat, making it hard to reach internal tissues. Hence, Fu et al. created a photoelectric-responsive Bi_2_S_3_/HAp nanorod array film on artificial Ti implants (Ti-BS/HAp) [105]. The composite structure of BS and HAp increased the NIR light absorption efficiency and the yield of photoelectrons. Upon NIR irradiation at 0.29 W cm^−2^, the photocurrent reached 25 μA cm^−2^, compared to 4 μA cm^−2^ for Ti-BS under the same condition. This photoelectric-responsive coating facilitated the control of cell fate to orient the osteogenic differentiation in vitro and was also able to enhance bone regeneration in vivo.

## Electroactive Hybrid Biomaterials in Bone Therapeutics Application

**Fig. 4:**
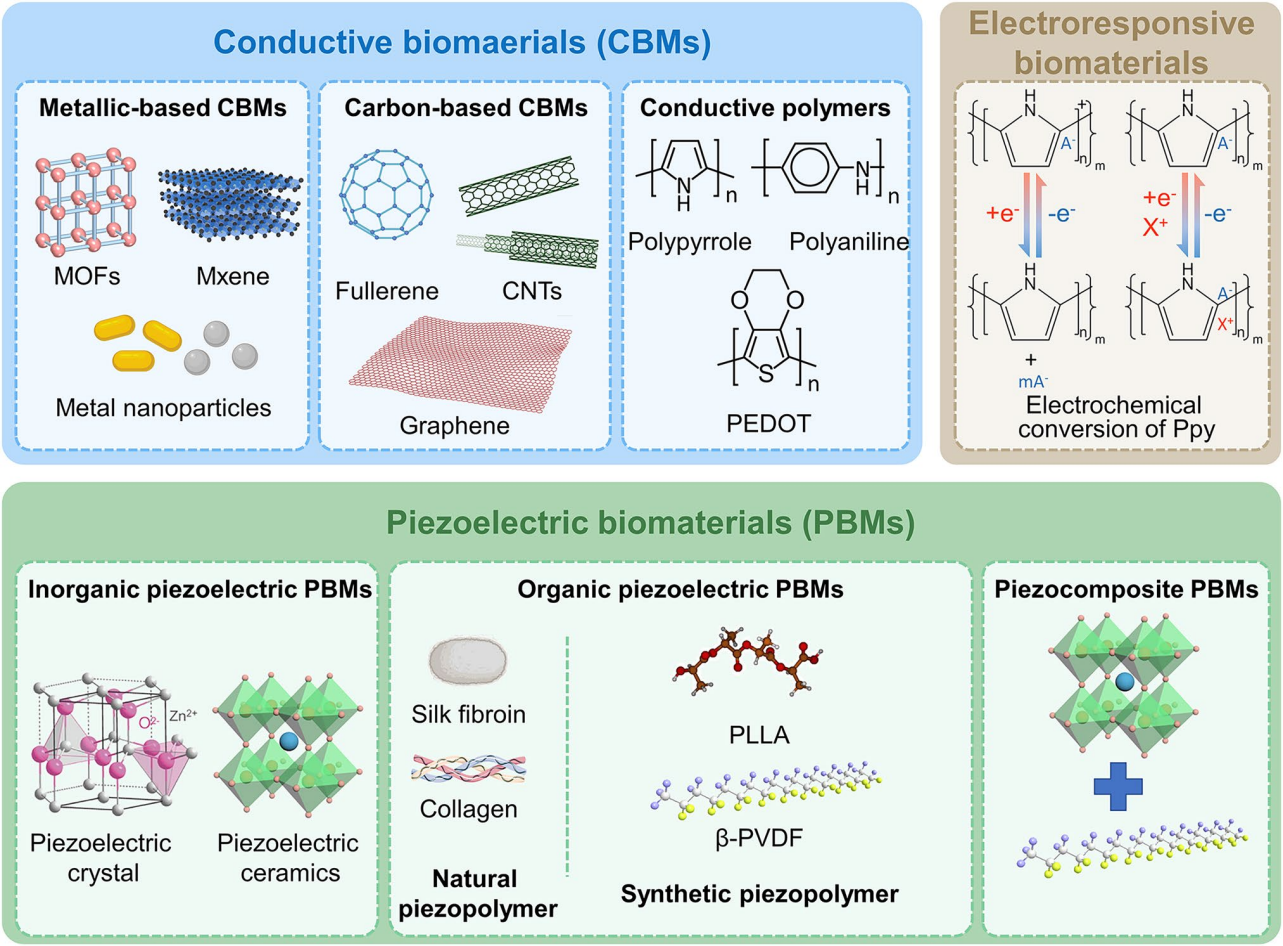
Typical electroactive hybrid biomaterials are applied for bone tissue regeneration, including conductive biomaterials and piezoelectric biomaterials

Owing to the discovery of bioelectricity and piezoelectricity, EHBs that simulate the electrophysiological microenvironment have attracted huge attention due to their capability to simulate Endo-EFs with high biocompatibility and low cost. Enormous efforts have been devoted to designing and fabricating EHBs (Fig. 4). By adjusting the components, topography, and structure, the properties of electroactive materials can be precisely customized to meet application requirements in different pathological conditions and maximize their potential value in bone tissue engineering.

### Electroactive Hybrid Biomaterials Classification

For the structure and properties of different types of electroactive biomaterials and their application in different scenarios in the field of bone repair and regeneration, as well as their advantages and disadvantages as bone repair materials, we summarized conductive biomaterials and piezoelectric biomaterials from the aspects of material structure and physical and chemical biological properties.

#### Conductive Biomaterials

CBMs can facilitate the regulation of cell–cell or cell–ECM cross talk by enhancing electron transport at the interface between bone cells or tissue and materials. The biological effects of CBMs on cell behaviors, including adhesion, migration, proliferation, and differentiation, have been extensively investigated [106]. Additionally, some reviews have summarized the fabrication of CBMs-based scaffolds with suitable biophysical cues (e.g., patterned morphology, micro/nanostructure, stiffness, and conductivity) for mimicking the properties of ECM and the stem cell niche [107, 108].

*Metallic CBMs* Traditionally, bulk metals such as titanium (Ti), cobalt (Co), stainless steel, and their alloys have been widely used as bone implants in clinical treatment due to their biocompatibility, excellent mechanical strength, and strong corrosion resistance comparable to natural bone tissue. However, these orthopedic metal implants are unable to degrade when applied in vivo, and the induced mechanical shunt may cause bone loss and even delay the healing process [109]. Hence, much attention has been paid to absorbable metal families, including magnesium (Mg), zinc (Zn), and iron (Fe), which have no concern about long-term fatigue damage and second surgery. Additionally, porous metal scaffolds have also been manufactured to meet the challenging requirements for bone implants. Their interconnected porous structure effectively increases the surface area for reinforcing osteoblast adhesion and proliferation [110]. The current application of metal implants for bone tissue treatment mainly relies on mechanical performance rather than electrical properties, so we will not discuss this in detail here.

*Metal nanoparticles* Represented by gold nanoparticles (AuNPs) and silver nanoparticles (AgNPs), these incorporate the outstanding conductivity and thermal properties of nanomaterials with exceptional physiochemical properties. They are generally utilized as dopants or coatings in preparing electroactive hybrid materials. AuNPs exhibit multifunctionality in modulating osteogenic cells and bone tissue. On the one hand, their controllable size and high surface area make them easy for surface modification or drug loading via gold-thiol chemistry to achieve eminent therapeutic potential. Reports have also revealed that AuNPs internalized by cells through endocytosis bind to proteins and generate mechanical forces on the cells, subsequently initiating the activation of the MAPK pathway, upregulating the expression of RUNX2, and downregulating PPARγ for the osteogenic differentiation of stem cells [111].

*MXene nanosheets* Composed of carbides, nitrides, or carbonitrides, MXene has rapidly emerged as a promising 2D nanomaterial potentially applicable in biomedical engineering [112]. MXene displays high volume capacitance, high conductivity, flexibility, and high surface area resulting from its stacked layered nanostructure. The excellent electroconductivity of MXene may result from three aspects. Primarily, MXene comprises transition metals that possess intrinsic conductivity. Second, the overlapping atomic orbitals within MXene layers provide a favorable environment for electron delocalization, while the layered structure increases the surface area. Simultaneously, the presence of carbon and nitrogen atoms between layers limits electron scattering, thereby promoting electron mobility and efficient charge transfer [113].

*Carbon-based CBMs* Carbon-based nanomaterials are nanoscale materials composed of carbon atoms, including carbon nanotubes (CNTs), graphene, and fullerene, among others [114–116]. They combine many superior properties, including inimitable mechanical features, chemical stability, large specific surface area, and high electrical conductivity, offering encouraging prospects for bone regeneration applications. Similar to metal nanoparticles and MXene nanosheets, carbon-based nanomaterials are usually doped into polymer matrices to facilitate the high conductivity and controllable mechanical properties of biomaterials. Among them, CNTs and graphene are two of the most commonly used carbon-based materials for bone tissue engineering. CNTs are equipped with nanotube structures formed by carbon atoms in a specific arrangement, with high stability of carbon–carbon bonds inside, giving them high flexibility and tensile strength. Peng et al. provided a detailed method to fabricate aligned CNT sheet-based artificial ligaments assembled with an anisotropic structure, allowing new bone regeneration and efficient repair of the bone tunnel [117]. Graphene exhibits excellent in-plane conductivity, enhancing electron transport within the plane [118]. Two derivatives, graphene oxide (GO) and reduced GO [119], have gradually developed with distinct conductivity from graphene. Specifically, GO is less conductive than graphene itself because GO introduces oxygen atoms and functional groups during the oxidation process, forming a carbon–carbon sp3 hybrid structure with oxidized functional groups [120].

*Conductive polymers* Compared to carbon-based materials and metals, conductive polymers (CPs) possess unique conductivity and exhibit characteristics typical of general polymers, especially flexibility and low stiffness. This combined property of CPs has aroused considerable research for modulating functions and repairing damage in various tissues, especially soft tissues involving the brain, heart, nerves, skin, and muscle [119, 121–123]. Notably, CPs can be designed and customized into injectable polymers to perfectly fit irregularly shaped bone defect areas, achieving precise regional bone regeneration. The charge conduction ability of conductive polymers arises from the hopping of electrons within and between polymer chains [113, 120]. CPs possess unique reversible doping characteristics in their redox states. It has been shown that doping performed at oxidation (p-doping) or reduction (n-doping) significantly increases a polymer’s electrical conductivity, varying from less than 10^–6^ S cm^−1^ in the neutral state to over 10^5^ S^−1^ cm in the doped state [124]. This capability endows CPs with capturing and controlling the release of bioagents, assisting in expanding the application of biosensors, bioprobes, and pharmaceuticals, thereby providing a variety of disease treatment options. Representative conducting polymers commonly used in cell stimulation and bone tissue regeneration mainly include polypyrrole (PPy) [125, 126], polyaniline (PANI) [127], poly(3,4-ethylenedioxythiophene) (PEDOT) [128], which also serve as appealing candidates for preparing electroactive scaffolds. Notably, PPy is also a typical electroresponsive material that can be reversibly switched between nanotubes and nanotips via electrochemical reduction/oxidation. Morphological switching will induce a highly adhesive hydrophobic surface to a poorly adhesive hydrophilic surface and vice versa, which can dynamically regulate the osteogenic differentiation of stem cells [126]. In its oxidized state, its carbon backbone enriches abundant positive charges and can serve as an active site for binding negatively charged drugs. Therefore, PPy has also become a promising candidate for constructing electroresponsive drug-release systems [129].

#### Piezoelectric Biomaterials

Piezoelectric biomaterials (PBMs) are a specific class of intelligent biomaterials due to their unique mechano-electro conversion capability, which can respond to mechanical stress (cell traction, body movement, tissue motions, ultrasound vibration, extension, and compression) and convert it into electricity and vice versa. The crystal structure and asymmetry of piezoelectric materials enable them to mimic the piezoelectric activity of bone tissue, promoting cell recognition and interaction with the material, and thereby initiating the proliferation and differentiation processes of bone cells. Additionally, piezoelectric materials generate charges along the stress direction due to charge distortion, providing ES that regulates ion diffusion in bone cells and body fluids. This modulation helps control cell signaling pathways, adjusting the function and activity of bone cells. This biomimetic design and biological similarity make PBMs promising and emerging materials in bone tissue engineering, providing strong support for innovative bone tissue repair strategies. Based on composition, piezoelectric materials will be discussed in three categories: inorganic PBMs, organic PBMs, and piezocomposites (Fig. 4).

*Inorganic PBMs* Inorganic PBMs are generally referred to as piezoceramics and crystals. Piezoelectric crystals such as quartz exist in nature in the form of single crystals, exhibiting limited piezoelectricity and reduced dielectric constant. In contrast, most piezoceramics display relatively high piezoelectric coefficients. Lead zirconate titanate (PZT), typically in lead-containing ceramics, exhibits a high piezoelectric coefficient of up to 200–350 pC N^−1^, remarkably expanding its wide application in electronics [130]. However, the biocompatibility issue cannot be ignored when applied to biomedical tissue engineering due to the possible release of toxic Pb^2+^ ions. In this way, the exploration and investigation of lead-free piezoceramics have become a research hotspot, mainly falling into three categories: perovskite structure, tungsten bronze structure, and bismuth layer structure [26]. Among them, perovskite-structured ceramics in an ABO_3_ chemical formula, such as BaTiO_3_ [131], SrTiO_3_ [132], LiTaO_3_ [133], LiNbO_3_ [134], BiFeO_3_ [135], and KNN [136], exhibit excellent biocompatibility and high electromechanical coupling [137]. Notably, BTO is the first piezoelectric ceramic used for bone tissue regeneration. BaTiO_3_ has an asymmetric tetragonal crystalline structure with its unit cell consisting of single Ti^4+^ and single Ba^2+^, where Ti^4+^ is located at the center of the unit cell and is covalently bonded to oxygen ions to form [TiO_6_] octahedra with barium ions at the four corners. Once exposed to mechanical stress, the BaTiO_3_ crystal experiences strong electrical polarity due to the shift of Ti^4+^ within every tetragonal unit cell [138]. When barium titanate is a tetragonal perovskite structure, the Ti atom shifts along the C-axis and the spontaneous polarization intensity along the C-axis, resulting in a decrease in crystal symmetry and a significant increase in piezoelectric property. Extensive studies indicate that BaTiO_3_ can induce the electrophysiological environment required for bone regeneration by surface polarization, thereby boosting the therapeutic potential for bone defects [139, 140]. The use of low-temperature process of perovskite-based hybrid biomaterial, in comparison with high-temperature treated ones, possesses better compatibility with flexible substrates and is more convenient for industrial commercialization. BaTiO_3_/Ti_6_Al_4_V (BT/Ti) scaffolds obtained at the low temperature effectively controlled the metabolism of macrophages and accelerate bone regeneration [141]. Moreover, the SrTiO_3_/Na_2_Ti_3_O_7_ coatings on Ti, which was produced through alkaline etching at the low temperature, showed great osteogenic capacity and significant potential in the field of hard-tissue implants [142].

*Organic PBMs* Distinct from the high rigidity and brittleness of inorganic piezoceramics, organic piezoelectric polymers exhibit better biocompatibility, flexibility, and lightweight, although they have relatively lower piezoelectric coefficients (− 10^–10^ pC N^−1^) than piezoceramics (100–3000 pC N^−1^) [143]. Moreover, the micro/nanostructure and mechanical properties of piezoelectric polymers can be precisely controlled and tailored through various fabrication techniques and easy processing methods, imparting them with exciting potential in biomedicine. Organic PBMs can be of natural or synthetic origin. Natural PBMs, including cellulose, glycine, collagen, silk fibroin, and chitosan, intrinsically possess biodegradability and bioactivity, thereby augmenting the cross talk between materials and bone tissue and fostering bone healing. Synthetic PBMs used for tissue regeneration mainly focus on PLLA [1], PVDF [144], and its copolymers (P(VDF-TrFE)) [145]. They present superior properties such as customizable mechanical and physical properties coupled with structural stability compared to natural PBMs. The piezoelectricity of piezoelectric polymers originates from the non-central symmetry and polarized molecular chain arrangement. PVDF and P(VDF-TrFE) are the most investigated piezoelectric polymers in bone tissue engineering due to their high flexibility and piezoelectricity, with piezoelectric coefficients of 20 and 30 pC N^−1^, respectively [146, 147]. Among the five crystalline phases (α, β, γ, δ, ε) of PVDF, the *β* phase displays the highest piezoelectricity (d_33_ = − 33 pC N^−1^; d_31_ = 23 pC N^−1^) [100], which results from the electronegativity difference between fluorine (F) and hydrogen (H) atoms, producing dipole moments in the F toward H direction in the –CH_2_–CF_2_– monomer [143]. In the case of PLLA, it is another commonly explored piezoelectric polymer, which exhibits remarkable biosafety and biodegradability than PVDF. The piezoelectric properties are similar to those of human bones, with a shear piezoelectric coefficient of − 9.8 pC N^−1^ [56].

*Piezocomposites* As mentioned earlier, bone is a composite of an inorganic mineralized matrix and an organic fibrous network that provides dynamic loading and sensing properties. This concept can inspire the preparation of composite scaffolds that integrate inorganic piezoelectric ceramics and organic piezoelectric polymers. Piezoelectric composite materials exhibit properties different from individual components, as they can address the high rigidity and brittleness of piezoelectric ceramics while significantly enhancing the low piezoelectric coefficient of a single polymer [56, 143]. Furthermore, the addition of certain conductive materials can serve as nucleating agents to promote the oriented alignment of molecular chain dipoles within piezoelectric polymers [148]. This allows the material to possess conductivity, flexibility, high piezoelectricity, and suitable mechanical strength, expanding the wide application of such materials in bone tissue regeneration.

### Application in Bone Therapeutics

Bone healing typically goes through the following four consecutive stages: hematoma formation phase, fibrous callus formation phase, bone formation phase, and bone remodeling phase [120]. This lengthy process involves the coordination of various physiological processes, including inflammation suppression, immune regulation, angiogenesis, and osteogenesis. Accelerating the bone remodeling process can be achieved through unilateral or systemic benign interventions using EHBs with good biocompatibility and functional diversity. Furthermore, EHBs can also serve as delivery platforms for therapeutic drugs or sensors for assisting in treatment and monitoring prognosis.

#### Osteogenesis Promotion and Osteoclast Suppression

Normal bone tissue is permanently in a highly dynamic and balanced state between bone formation mediated by osteoblasts and bone resorption mediated by osteoclasts [120]. The activity of osteoblasts is associated with the synthesis of bone collagen and mineralization of bone tissue, while osteoclasts are responsible for breaking down and clearing necrotic or aging bone cells. Multiple cells and cytokines collectively regulate bone homeostasis through a complex signaling network. When this balance is disrupted, various types of bone-related diseases will occur unexpectedly. Osteoporosis is caused by the abnormal excessive activity of osteoclasts, leading to a bone resorption rate that exceeds the rate of bone formation by osteoblasts [149]. Therefore, utilizing EHBs to suppress osteoclast activation while synergistically enhancing osteogenesis will be a promising strategy for disease treatment. For example, Li’s group proposed a tricalcium phosphate (TCP)-based gelatin scaffold (GGT) in combination with electroacupuncture stimulation to study the ES effect on the activation of osteoclasts and osteoblasts [150]. In vitro assays revealed that ES simultaneously activated osteoblasts and osteoclasts via inducing pH changes, while they also found that a suitable ES of 200 Hz/1 mA promoted osteoclast activity in the early status of bone remodeling and inhibited its differentiation in the bone formation stage. Compared with other current methods for the treatment of osteoporosis or bone regeneration, the combination of TCP-based gelatin scaffolds and ES has a better biosafety effect and can better promote bone regeneration [150]. Cui et al. created a zoledronic acid (ZA)-loaded piezoelectric PLLA coating on the Ti-based bone implant via electrospinning technology to prevent aseptic loosening for the first time [151]. The porous microstructure of the fibrous coating inhibited the activity of osteoclasts through the long-term release of ZA. Additionally, the ZA drug served as a nucleating agent to significantly enhance the piezoelectricity of the PLLA/ZA coating. Under in situ ES triggered by cell traction force during adhesion and migration, the PLLA/ZA coating promoted MSCs osteogenesis and osseointegration after implantation in femur defects. Additionally, the spontaneous polarization and surface charges of piezoelectric materials can also change the adsorption amount or conformation of proteins, exposing or masking structural domain sequences that bind to cells, thereby regulating cell adhesion and migration behavior [152, 153]. In one study, the surface potential of nanocomposite membranes composed of polydopamine (PDA)-coated BATiO_3_ and P(VDF-TrFE) matrix could be tuned up to  − 76.8 mV by optimizing the composition ratio and corona poling treatment, conforming to the level of endogenous biopotential [154]. This composite membrane maintained the electrical environment and encouraged mature bone formation.

#### Immunomodulation

**Fig. 5:**
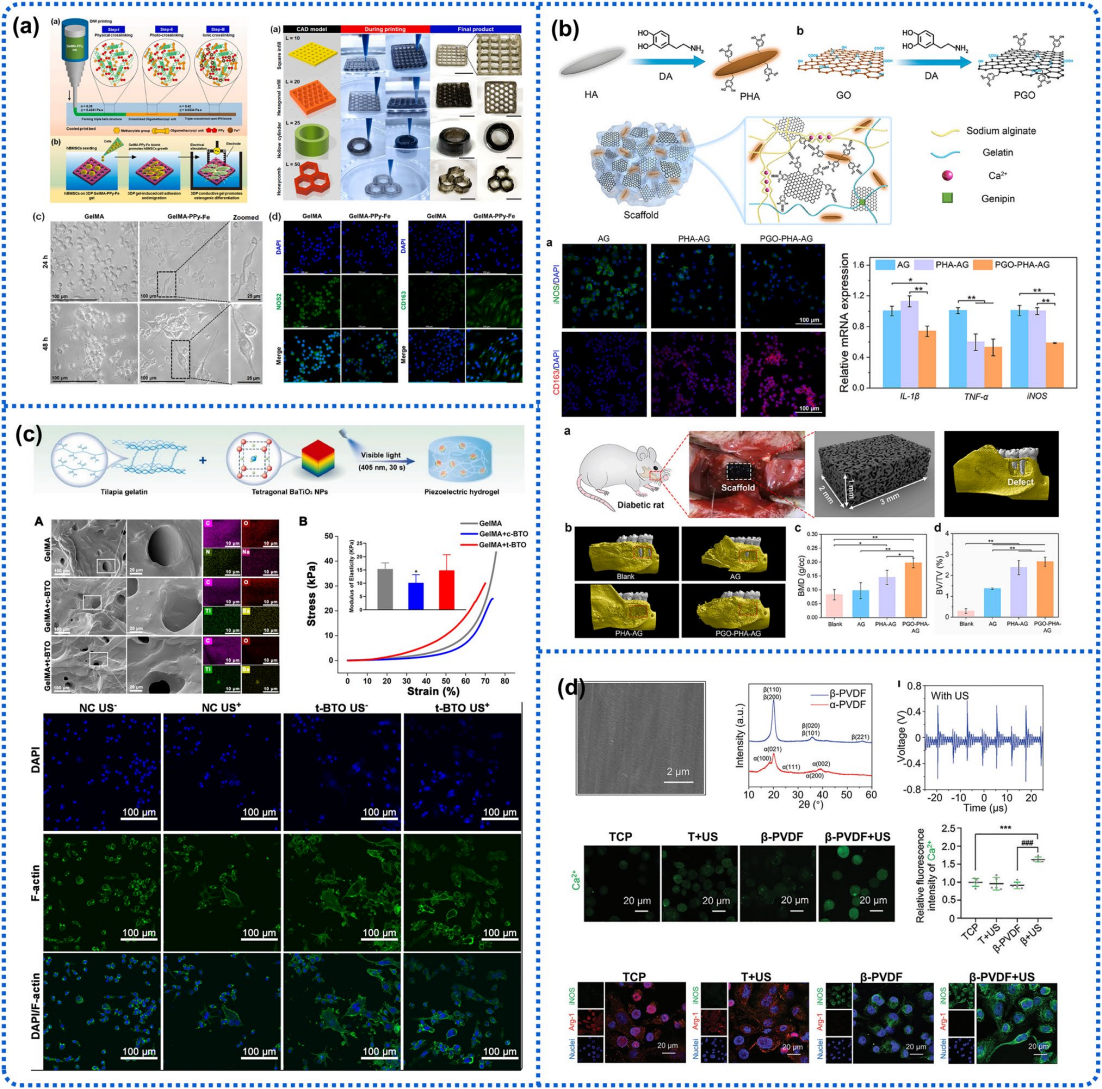
Effect of immunomodulation for electroactive hybrid biomaterials for macrophage polarization and bone regeneration. **a** Fabrication of conductive GelMA/PPy 3D-printing scaffold and its positive effects on osteogenesis via collective signaling and immunopolarization. Reproduced with permission [157]. Copyright 2023, Elsevier. **b** Immunomodulatory ability of PGO-PHA-AG scaffolds for periodontal bone regeneration in diabetes. Reproduced with permission [115]. Copyright 2022, Elsevier. **c** Switching of macrophage phenotype induced by the US-stimulated ES through Injectable piezoelectric hydrogels. Reproduced with permission [160]. Copyright 2024, Elsevier. **d** Regulation of pro-inflammatory macrophage polarization through PVDF-mediated wireless localized ES. Reproduced with permission [161]. Copyright 2021, Wiley–VCH

The immune system plays a key role in the development and progression of bone-related diseases; its interaction with bone cells is pivotal for both bone homeostasis and pathology [155]. Studies have shown that various immune cells, especially macrophages, can secrete related cytokines directly influencing bone immune responses and regenerative repair. In addition to chemical-based methods, ES-induced physical approaches have been confirmed to implement immunoregulatory effects, which may positively influence anti-inflammation and facilitate bone healing [156, 157]. It has been reported that ES can promote the migration and proliferation of immune cells such as lymphocytes, neutrophils, and T cells [158, 159]. Macrophages, exhibiting two phenotypes (M1 pro-inflammatory and M2 anti-inflammatory), are an important class of multifunctional immune cells that play key roles in tissue repair, host defense, and homeostasis. Up to now, EHBs have been developed to inhibit the continuous release of inflammatory and chemotactic factors by repelling the pro-inflammatory phenotype of macrophages, thereby promoting tissue repair [157]. In one study, a conductive PPy-grafted gelatin methacryloyl (GelMA-PPy) scaffold fabricated by 3D-bioprinting was observed to boost the osteogenic potential of BMSCs under direct electrostimulation (250 mV/20 min/day) [157], attributed to the positive effect on immunopolarization of M2 macrophages together with the modulation of NOTCH/MAPK/SMAD signaling and Wnt/β-catenin pathways (Fig. 5a). Similarly, Li et al. also created hydrogel-based scaffolds composed of PDA-modified GO (PGO) and HAp nanoparticles (PHA) introduced into alginate/gelatin scaffold (PGO-PHA-AG), enhancing periodontal bone regeneration in a diabetic rat model by modulating the inflammatory microenvironment through interrupting glycolytic and RhoA/ROCK pathways (Fig. 5b). Catechol groups on the scaffold primarily reduced the inflammatory state of bone defects, thus increasing cell survival. Without additional ES, the conductive PGO-PHA-AG facilitated the immunoregulation of bone regeneration by activating M2 macrophages and secreting osteogenesis-related factors [115].

Recently, piezoelectric biomaterials have emerged as a promising platform for macrophage polarization. Wan et al. proposed a piezoelectric composite hydrogel comprising tetragonal BTO nanoparticles and GelMA [160]. They found that the hydrogel-generated piezopotential recovered the osteogenic capability of inflammatory periodontal ligament stem cells (PDLSCs) by lowering mitochondrial membrane potential and promoting adenosine triphosphate (ATP) synthesis for the first time (Fig. 5c). This scaffold reshaped an anti-inflammatory and pro-regenerative niche by M2 phenotypic polarization, rescuing osteogenesis, and dramatically enhancing in situ bone regeneration. An in vitro study reported by Liu et al. indicated that the US-triggered piezoelectric charges on the surface PVDF film remarkably enhanced the M1 polarization of macrophages [161]. The localized electrical signals enabled Ca^2+^ influx through voltage-gated channels and the Ca^2+^-CAMK2A-NF-κB axis, thereby promoting the release of the pro-inflammatory factors TNF-*α* and IL-1*β* (Fig. 5d).

#### Angiogenesis Enhancement

**Fig. 6:**
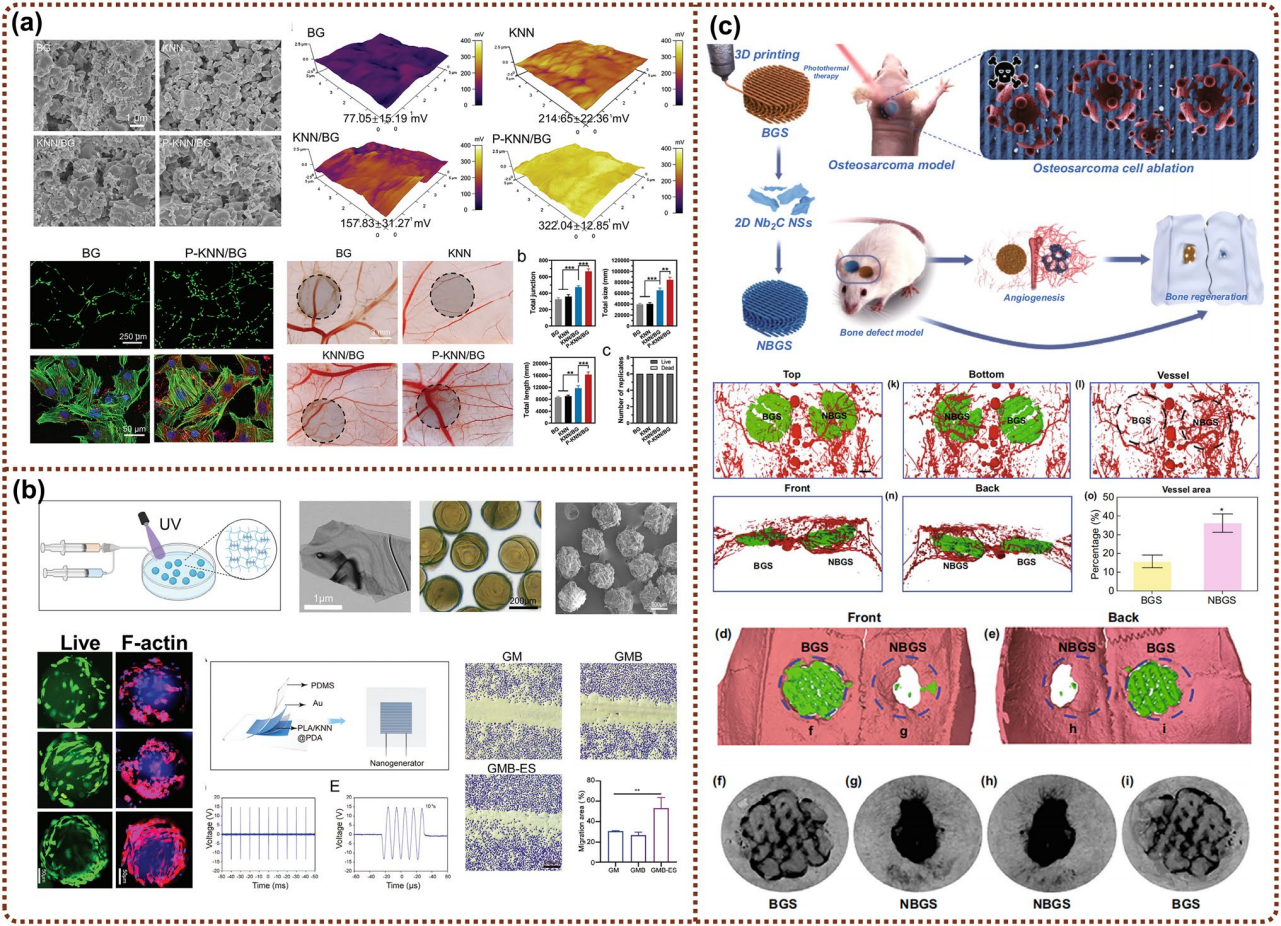
Electroactive hybrid biomaterials for accelerating bone regeneration through enhanced angiogenesis. **a** Morphology and surface potential of piezoelectric bioactive glass films and their angiogenesis effects. Reproduced with permission [169]. Copyright 2023, Wiley–VCH. **b** Immuno-angiogenic dual functions of cell-loaded conductive GelMA hydrogel microspheres. Reproduced with permission [170]. Copyright 2024, Wiley–VCH. **c** MXene‑functionalized scaffolds for osteosarcoma phototherapy and angiogenesis/osteogenesis of bone defects. Reproduced with permission [171]. Copyright 2021, Springer

Blood vessels are an integral part of the skeletal system and essential for maintaining bone homeostasis [162]. The vascular network not only transports nutrition and oxygen for bone tissue metabolism but also offers a stable niche for the self-renewal and differentiation of bone cells. During the healing process of bone defects, insufficient angiogenesis caused by the implantation of bone repair materials is an issue that urgently needs to be addressed, as it can negatively result in nonunion and avascular necrosis, ultimately increasing the risk of bone defect reconstruction failure [163–165]. Many researchers have illustrated that ES can induce endothelial cell migration and enhance the secretion of epidermal growth factor [166] and vascular endothelial growth factor (VEGF) to promote healing metabolism [59, 167, 168]. Current studies have shown that EHBs can reproduce the bioelectrical microenvironment, thereby promoting angiogenesis by mediating relevant metabolic pathways to accelerate bone tissue vascular formation and bone repair. Wang et al. constructed a piezoelectric bioactive glass composite based on polarized potassium sodium niobate (P-KNN/BG) to directly induce angiogenesis (Fig. 6a) [169]. The piezoelectric P-KNN/BG exhibited a positive effect on HUVECs adhesion, migration, proliferation, and especially angiogenesis, as reflected by increasing tube formation and expression of angiogenesis-related growth factors through activating the eNOS/NO signaling pathway. In vivo results further demonstrated the pro-angiogenic effect of piezoelectric P-KNN/BG by using a chick chorioallantoic membrane model. In another study, a novel dental pulp stem cell (DPSC)-loaded conductive GelMA hydrogel microspheres integrated with black phosphorus (BP) nanosheets were fabricated, and the wireless ES was supported by a US-driven biodegradable PENG (PLA/KNN@PDA) [170]. The angiogenic behavior could be improved on a DPSC-mediated paracrine pattern by the fine-tuned electric signal, ultimately establishing an immuno-angiogenic niche at an early stage of tissue repair and promoting bone defect regeneration (Fig. 6b).

In addition, EHBs can also promote vascularization for bone tumor treatment. Yin and coworkers proposed a photonic-responsive two-dimensional (2D) ultrathin niobium carbide (Nb_2_C) MXene nanosheets (NSs) into 3D-printed bioactive glass scaffolds (NBGS) for the multi-effect treatment of osteosarcoma [171]. The introduction of the Nb element promoted the neogenesis and migration of blood vessels in the defect site, thus ensuring the transport of more oxygen, vitamins, and energy around the bone defect for the reparative process (Fig. 6c).

#### Anti-Bacterial Effect

**Fig. 7:**
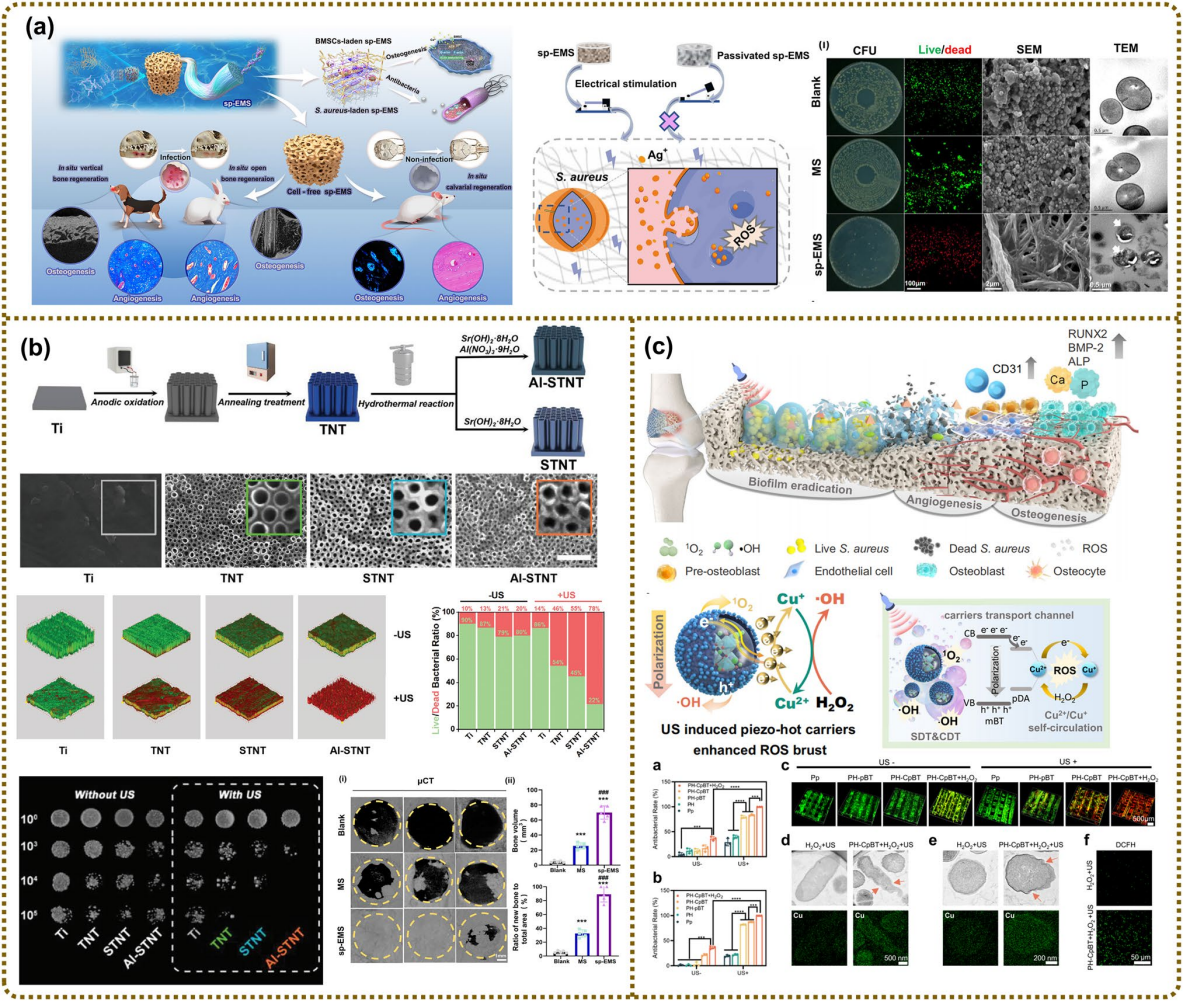
Designing electroactive hybrid biomaterials with antibacterial and osteointegration properties for bone remodeling. **a** Self-promoted electroactive biomimetic mineralized scaffolds for bacteria-infected bone regeneration. Reproduced with permission [174]. Copyright 2023, Nature. **b** Piezo-sonocatalytic nanocoating on dental implants with integrated antibacterial performances and osteogenic activity. Reproduced with permission [175]. Copyright 2024, Wiley–VCH. **c** Multifunctional nanoreactor composed of BaTiO_3_ and copper inducing cuproptosis-like bacterial death against implant infections. Reproduced with permission [183]. Copyright 2024, Nature

Bacteria, as electrically responsive microorganisms, typically carry a negative charge under normal circumstances. When exposed to a positively charged environment, the negative charges on the bacterial membrane can be easily neutralized, changing the structure of the lipid bilayer, increasing membrane permeability, and ultimately leading to bacterial penetration and rupture [172, 173]. Inspired by this, the exploration of EHBs provides a feasible approach for infected bone regeneration. EHBs with antimicrobial properties can particularly promote the healing and regeneration of bone tissue under specific pathological conditions, such as periodontitis, arthritis, or the biofilm issue in clinical bone implants [174–182]. Li et al. reported a self-promoted electroactive mineralized scaffold (sp-EMS) obtained by intrafibrillar mineralization of the co-assembled AgNWs with collagen molecules [174]. The sp-EMS potentiated bactericidal effects by self-promoted electrostimulation, synergistically inhibiting bacterial adhesion and completely breaking down the membrane (Fig. 7a). As an application of the concept, the sp-EMS equipped with osteogenic and antibacterial dual functions successfully realized nearly complete in situ bone regeneration in rats with single bacterial infections, even regenerating in situ rabbit open bone defects and dog vertical bone defects in a complex bacterial microenvironment [174]. In a different study, Lin et al. designed a US-responsive piezoelectric nanocoating composed of Al ion-doped strontium titanate/titanium dioxide nanotubes (Al–SrTiO_3_/TiO_2_ nanotubes, Al-STNT) on the surface of a Ti dental implant (Fig. 7b). The doping Al^3+^ introduces oxygen vacancies into the SrTiO_3_/TiO_2_ heterojunction, while the US-stimulated piezoelectric effect assisted in overcoming the bandgap barrier to generate more reactive oxygen species (ROS), thereby reinforcing the antibacterial performance and supporting the potential use of Al-STNT in the treatment of peri-implant infections [175]. Similarly, a PDA-modified BTO sonosensitizer chelated with copper (CpBT) was fabricated and incorporated into polyetherketoneketone (PEKK) [176] implants to amplify the efficacy of sonodynamic and chemodynamic therapy against implant infections (Fig. 7c) [183]. The high-efficiency antibacterial properties mainly resulted from overloaded intracellular Cu^2+^ and elevated ROS amounts caused by Cu^+^ -catalyzed chemodynamic reactions, ultimately promoting angiogenesis and osteogenesis on demand in vivo. We have compared several materials in this paper for antibacterial effect (Table 1).

**Table 1:** Comprehensive information on various materials for antibacterial effect

Materials	Bacteria	Mechanisms	References
Na_0.5_K_0.5_NbO_3_	S. aureus	Electrical stimulation	[177]
3D conductive PCL/TrGO scaffold	S. aureus	Electrical stimulation	[178]
Self-promoted electroactive mineralized scaffold	S. aureus	Electrical stimulation	[174]
Al–SrTiO_3_/TiO_2_ nanotubes	P. gingivalis, F. nucleatum	Inducing ROS	[175]
PDA-modified BTO sonosensitizer	–	Inducing ROS	[176]
Hydroxyapatite/barium titanate	E. coli, P. aeruginosa S. aureus	Charge neutralization	[179]
Zn–Cu alloy micro batteries/galvanic couples	S. aureus	Preventing the biofilm formation	[180]
PVDF piezoelectric surfaces coated with essential oil nanoparticles and acylase	P. aeruginosa S. aureus	the piezoelectric effect	[181]
Ag-doped mesoporous S_53_P_4_ bio-ceramics	S. aureus E. coli	–	[182]

## Self-Powered Bioelectronic Devices in Bone Therapeutics Application

As a non-pharmacological rehabilitation method, electronic sensing and stimulation at multiple sites throughout the body enable bioelectronic therapies that go far beyond what is possible with pharmaceuticals, substantially improving medical outcomes by regulating the activity of excitable cells or bone tissue, thereby promoting bone regeneration and accelerating functional recovery. Nevertheless, only a few bioelectronic devices meet the standard of care for clinical use, such as cardiac pacemakers, deep brain stimulation devices, and continuous glucose monitors [184, 185]. However, the further development of these devices is considerably restricted by large battery packs, cumbersome tethers, and intricate packaging [186]. Additionally, these traditional devices face risks of implantation infection, uncomfortable wearing, limited battery lifetime, and potential environmental toxicity [79]. To the best of our knowledge, no microelectronic devices are operating without a battery available in the clinic for the prevention, therapy, or postoperative monitoring of bone disease.

The urgent need for battery-free bioelectronic devices has spurred a series of innovations in self-powered materials and technologies to overcome the challenges of power requirements [84]. These include electromagnetic, magnetic, ultrasound, and optical methods. Additionally, piezoelectric, triboelectric, magnetoelastic, photovoltaic, and thermoelectric materials are being evaluated for use in next-generation energy harvesting. Among these emerging materials and technologies, TENGs and PENGs with excellent flexibility, portability, and highly efficient electromechanical conversion have stood out for their ability to receive and harvest the widely distributed and underutilized biomechanical energy in the human body. By optimizing the device architecture and package, TENG and PENG can realize miniaturization to millimeter-scale dimensions, ensuring adjustable, constant, and safe ES for disease treatment.

### TENGs for Bone Therapeutics

Bone fractures caused by high-force impacts or stress have made this common musculoskeletal disorder a critical problem in health and economics. As mentioned, the key to bone reconstruction lies in maintaining the balance between osteoblast differentiation and osteoclast resorption [187]. Compared to inhibiting the activity of osteoclasts, a more effective approach is to elevate the bone-forming ability of osteoblasts. The first investigation using TENGs to regulate osteoblast behavior was conducted by Tang et al., who reported a self-powered low-level laser cure (SPLC) system composed of a flexible arch-shaped TENGs and an infrared laser excitation unit for osteogenesis [188]. A pyramid array was patterned on the friction materials to optimize the TENG output, achieving a short-circuit current of 30 μA and an open-circuit voltage of 115 V under a displacement of 0.5 mm and a frequency of 50 Hz, successfully driving two laser groups. In vitro results displayed enhanced MTT absorbance, more mature ALP extracellular matrix, and significant mineral deposition after the TENG-laser treatment, suggesting the positive effect of the TENG-laser system on proliferation, differentiation, and bone formation when compared with the control group (no treatment). Additionally, the TENG-laser and battery-laser groups exhibited a comparable influence on osteoblast differentiation, although the cell proliferation effect of the battery-laser irradiation was slightly greater than that of the TENG-laser group. By fixing the entire SPLC system on a volunteer’s arm, this team confirmed that the TENG could generate durable electricity triggered by arm swings during walking to power the laser unit. Besides the wearable capability, the implantation potential of TENG (iTENG) was also demonstrated by inserting the iTENG between the liver and diaphragm of an adult rat. The breath-induced deformation driven by inhalation and exhalation contributed to a stable and continuous electric output of about 0.06 nA and 0.2 V. This study broadens the scope for the application of TENGs in portable or implantable self-powered medical devices, as well as for clinical therapy of osteoporosis and orthodontic treatment.

**Fig. 8:**
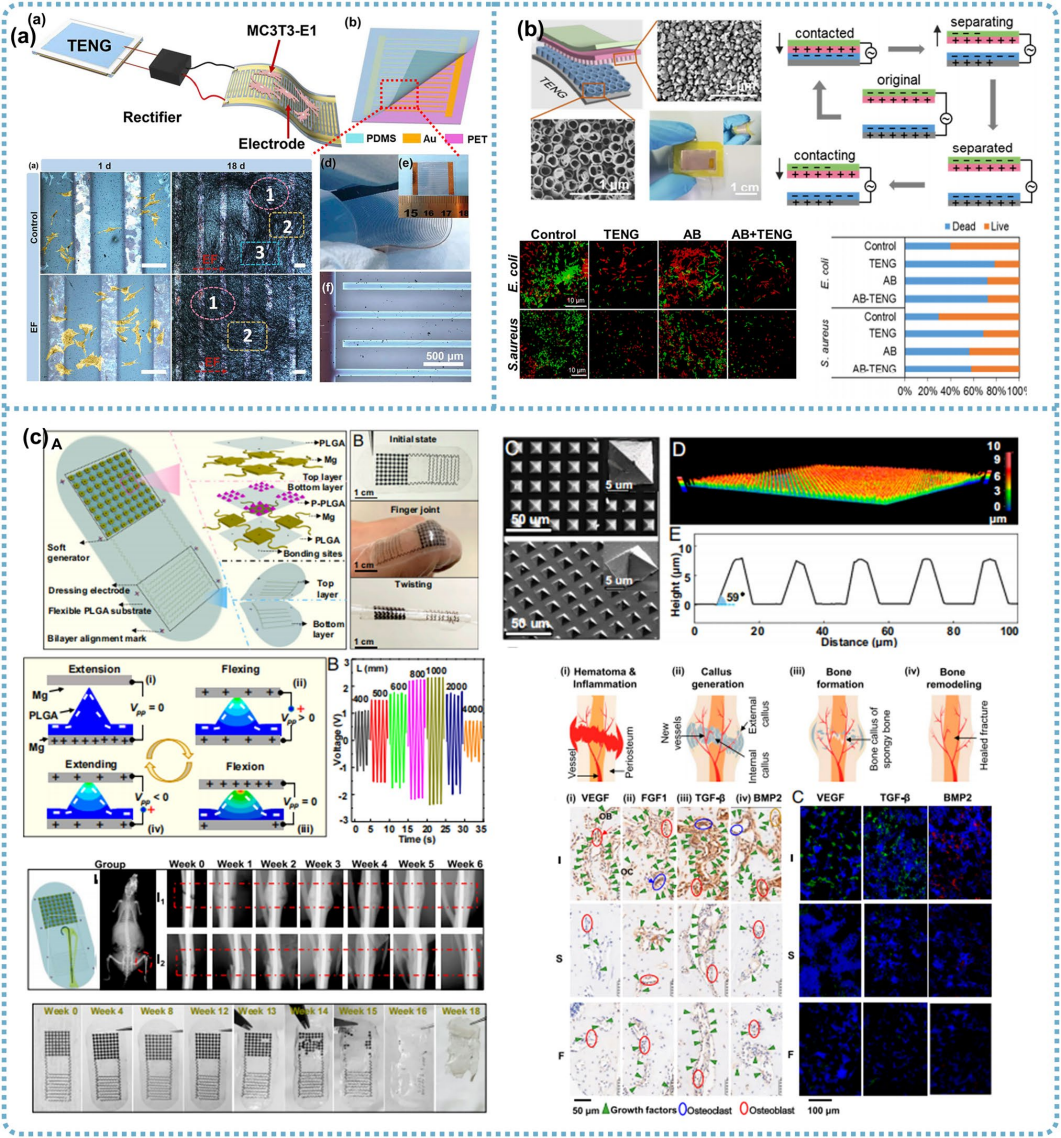
Self-powered TENG device for osteogenic regulation and bone regeneration. **a** Self-powered implantable TENG integrated with flexible electrode for osteoblast proliferation and differentiation. Reproduced with permission [89]. Copyright 2019, Elsevier. **b** A nanostructured-TENG fabricated to accumulate negative charges on Ti implant to inhibit biofilm. Reproduced with permission [95]. Copyright 2020, Elsevier. **c** A self-powered implantable and bioresorbable electrostimulation device for biofeedback bone fracture healing. Reproduced with permission [189]. Copyright 2021, National Academy of Sciences

In addition to powering therapeutic components, TENGs can directly intervene in the remodeling process of bone tissue by generating electrical stimulation [190, 191]. Tian et al. fabricated a self-powered implantable electrical stimulator by connecting a contact-separation-mode TENG with a flexible interdigitated electrode [89]. In vivo assessment was conducted by implanting the stimulator on the surface of the fracture region, and it was found that the device generated excellent and safe output (0.06 V, 1 nA) during the rat’s daily activity (Fig. 8a). Specifically, the ES signals induced by TENGs could directly deliver to MC3T3-E1 cells cultured on the electrode and enhance adhesion, proliferation, and osteogenic differentiation. After TENG-mediated ES, the intracellular Ca^2+^ level increased immediately from 51.0% to 64.1%, suggesting that cell behavior might be modulated through Ca^2+^ signal transduction activating ion channel-participated pathways [89]. Based on this research, the same team designed and prepared a TENG with a nanorod-like triboelectric layer on the nanotube-like surface of a Ti implant [95]. By harnessing human motion to drive the TENG, the generated negative charges sustainably and long-term accumulated on both sides of the implant (Fig. 8b). Therefore, bacterial adhesion and growth were effectively suppressed, ultimately preventing the formation of biofilms without introducing any side effects. With the assistance of antibacterial properties, the ES generated by TENG markedly promoted MC3T3-E1 adhesion, proliferation, and osteogenic differentiation. This work is one of the earliest to explore the multifunctionality of TENG to energize bone implants with anti-biofilm and osteogenesis promotion activity simultaneously, encouraging new design impetus for wearable or implantable TENGs in orthopedic or dental implants.

Different from the durability of wearable TENGs, the biocompatibility and bioabsorbability of implantable TENG used for bone tissue can effectively mitigate the adverse effects on the host and environmental pollution issues, as well as avoid infections induced by invasive surgical removal. Given these considerations, Zheng and his colleagues fabricated a fully biodegradable TENG (BD-TENG) electrostimulator with a multilayer structure, composed of biodegradable polymers forming the friction layer and resorbable Mg metal forming the electrode layer [190]. The whole BD-TENG was endowed with tunable degradation properties by sealing it into different encapsulations. As displayed in Fig. 8a, the BD-TENG encapsulated with poly(l-lactide-co-glycolide) (PLGA) maintained structural integrity even on day 40 and degraded on day 90. In contrast, the poly(vinyl alcohol) (PVA)-coated device experienced an ultratransient degradation process and completely dissolved within 72 h. This work creatively provides a reference for selecting materials for the preparation of TENG, and the controlled degradation rate ensures the device matches the therapeutic period of different diseases. Once therapy is completed, the TENG remaining in the body can be safely degraded and metabolized. In 2021, Wang’s team first demonstrated the feasibility of BD-TENG as ES therapy to accelerate bone fracture healing at the animal experiment level. They designed an ultraflexible, implantable, and biodegradable bone fracture ES device [192] and realized biofeedback ES by generating stable electric pulses in response to the movement of the knee joint [189]. In vivo study exhibited the duration of stable function for 8 weeks, enabling the device to provide sufficient ES intervention and achieve an excellent therapeutic effect in as short as 6 weeks. Figure 8b depicts the healing mechanism of the FED device. With the increasing secretion of cytokines (VEGF and FGF1) and proteins (TGF-β and BMP2), the vascularization for nutritional supply and metabolic transportation was remarkably enhanced, contributing to osteoblast differentiation, mineralization, and bone healing (Fig. 8c). Together, this study proposed a valuable concept as closed-loop self-powered ES for bone fracture healing, expected to significantly reduce the treatment cost and pain of patients.

**Fig. 9:**
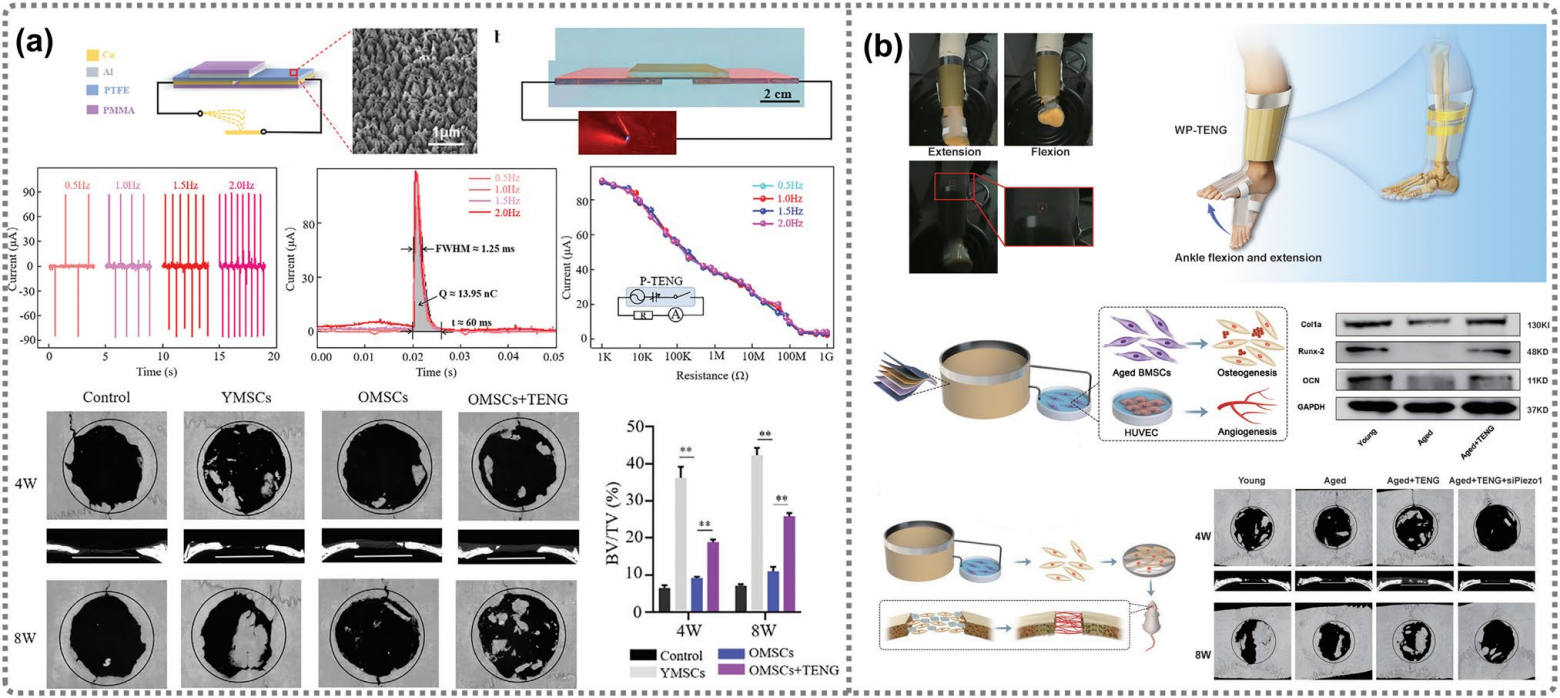
Self-Powered TENG for Alleviating Bone Aging. **a** Pulsed-TENG based on lateral sliding mode enhanced bone regeneration through the rejuvenation of senescent BMSCs. Reproduced with permission [144]. Copyright 2021, Wiley–VCH. **b** Wearable pulsed-PENG triggered by ankle motion for bone repair by rejuvenating aged BMSCs. Reproduced with permission [193]. Copyright 2021, Wiley–VCH

As a natural and inevitable physiological process, aging decreases bone density, and bone loss becomes increasingly common in the elderly population, thus raising the risk of osteoporosis and fractures [194]. Degeneration of cellular and tissue functions caused by aging, in turn, severely hinders bone repair and regeneration. One important factor is the senescence of bone marrow mesenchymal stem cells (BMSCs), which encounter dysfunction in osteogenic differentiation and induced inflammatory niches. Utilizing triboelectric technology as a promising therapeutic strategy, Wang et al. reported a pulsed-TENG (P-TENG) based on sliding mode to rejuvenate the old BMSCs (OMSCs) of aged human donors for the first time (Fig. 9a) [88]. After customizing the structure, P-TENG generated stable electric output unaffected by the changed frequency and produced a pulsed current of 30 μA at 1.5 Hz. This stimulation parameter was verified to alleviate the senescent phenotype of OMSCs by promoting proliferation and pluripotency. Importantly, TENG-mediated pulsed stimulation exhibited better biological compatibility compared with direct current. In an 8-week animal assay, the restoration of the osteogenic potential of OMSCs in vivo by P-TENG was further demonstrated by Micro-CT and immunohistochemistry. Creatively, this study revealed an essential role of the MDM2-p53 pathway in the rejuvenating effect of triboelectric stimulation on aged BMSCs. It is worth mentioning that systematic studies could be conducted to optimize the electrical parameters to realize the best therapeutic effect. To further implement the application potential of P-TENG, the same group updated the structure of P-TENG based on the previous prototype. Specifically, a wearable P-TENG (WP-TENG) was fabricated to reinforce the osteogenesis of aged BMSCs and promote the angiogenesis of HUVECs to collectively achieve bone repair and regeneration [193]. As displayed in Fig. 9b, the WP-TENG could be self-driven by ankle extension and flexion to generate ES directly delivered to the bone defect region. This research indicated the satisfactory effect of WP-TENG on rejuvenating aged BMSCs by enhancing osteogenesis ability and promoting vascular tubule formation. Mechanistically, triboelectric stimulation activated the mechanosensitive Piezo1 ion channel, promoted the influx of Ca^2+^, regulated the expression of the transcription factor HIF-1α, and thus, upregulated osteogenesis-related genes and increased the secretion of angiogenic factors. In general, two studies have displayed the encouraging potential of TENG in reversing the activity and function of aging BMSCs. Despite positive progress in animal assays and mechanism exploration, challenges and unsolved issues remain before clinical standards can be achieved.

### PENG for Bone Therapeutics

The working principle of PENGs relies on the piezoelectric effect, which is closely associated with the generation of electric dipole moments under deformation caused by the non-centrosymmetric nature of materials. Electric dipole moments can be induced by ions on asymmetric lattice sites, common in most piezoelectric ceramics (PZT, BTO, ZnO, etc.) [195]. They can also exist directly in molecular chain groups and orientations, typical of most biomacromolecules and piezoelectric polymers, such as collagen, DNA, PVDF, and PLLA. Due to their unique electromechanical conversion properties, electricity is generally produced once the piezoelectric materials experience deformation and vice versa. This is termed the direct piezoelectric effect and the converse piezoelectric effect, respectively.

The piezoelectric effect, resulting at the molecular level, objectively exists in piezoresponsive materials. External stimuli induce internal molecular dipole polarization and orientation, leading to the generation of a macroscopically visible piezoelectric potential [196]. Its presence allows piezoelectric materials to directly interface with living organisms, such as cells and tissues, serving as a determining cue in tissue regeneration. The relevant discussion has been elaborately stated in the previous sections, so we will not reiterate this part excessively here.

Similar to TENGs, PENGs can be explored as wearable devices with no interface with any components of bone tissue (cells, collagen, and tissue) or designed as implantable self-powered energy-harvesting or therapeutic devices [101]. Depending on the piezoelectric materials, especially water-soluble piezoelectric materials, the external humidity is not conducive to generating an effective electrical signal output through the static electricity caused by extrusion, which needs to rely on high-precision packaging.

**Fig. 10:**
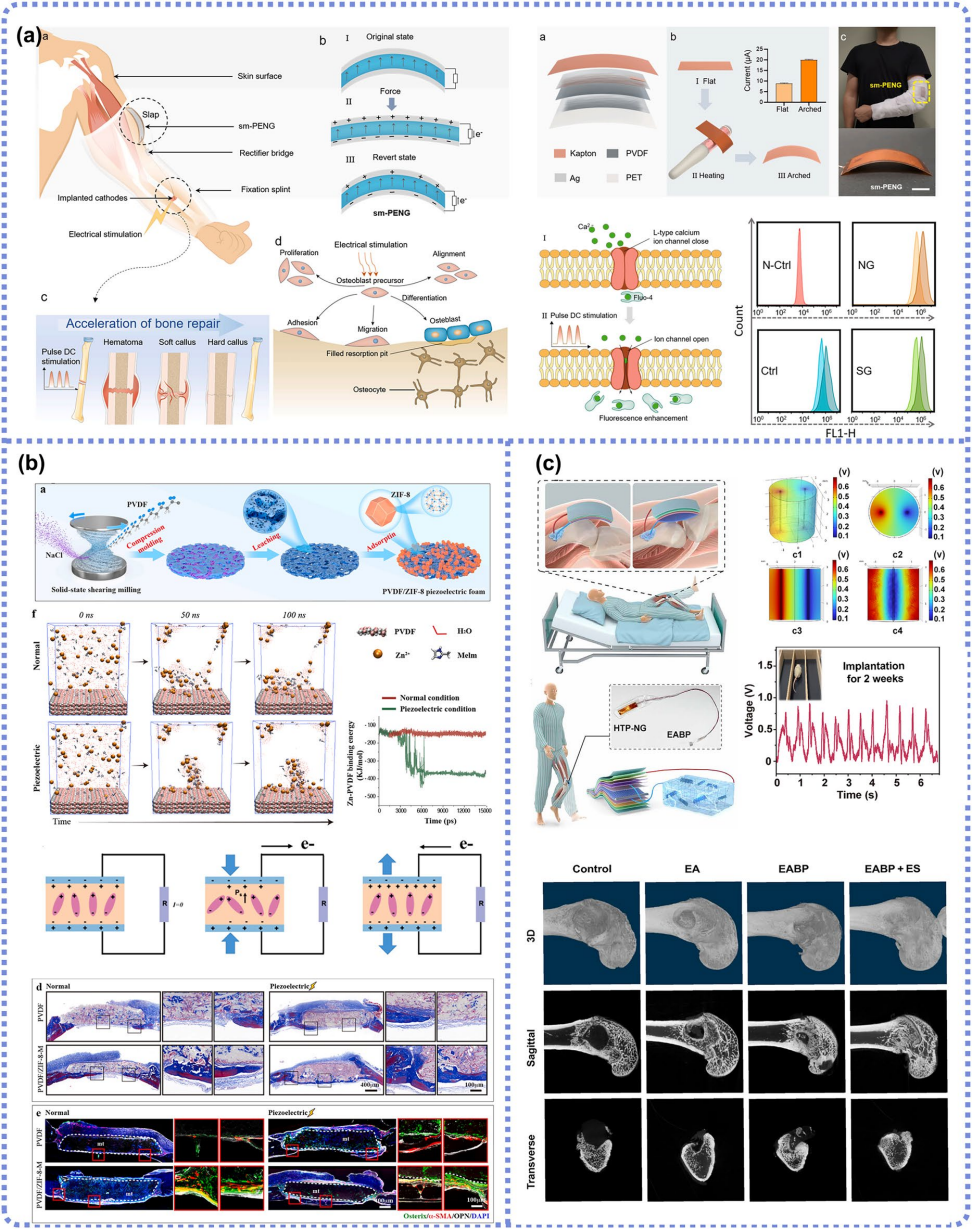
Self-Powered PENG for Bone Regeneration. **a** Fixation splint with biomechanical energy-driven shape memory piezoelectric nanogenerator (sm-PENG) to promote bone repair. Reproduced with permission [197]. Copyright 2021, Elsevier. **b** ZIF-8 decorated PVDF foam PENG for enhanced bone regeneration. Reproduced with permission [198]. Copyright 2023, Elsevier. **c** Symbiotic electrical stimulation system composed of hybrid PENG/TENG and conductive hydrogel electrode for accelerating bone regeneration during rehabilitation exercise. Reproduced with permission [199]. Copyright 2024, Science

However, everything has its pros and cons. The complex physiological environment and the stress-absorbing properties of bone tissue easily weaken the actual performance of PENGs, affecting their practical application. Therefore, the core of developing feasible PENGs lies in the selection of piezoelectric materials and the design of device structures. Utilizing material engineering techniques such as surface modifications and interfacial polarization can improve the responsiveness, thereby enhancing their electrical output performance for bone tissue engineering [200, 201]. From a structural optimization perspective, designing piezoelectric materials with different morphologies and structures, such as fibrous, arrayed, fabric-like, and porous, can enhance the mechanical performance of PENGs and improve their electromechanical conversion efficiency. Zhang et al. reported a biomechanical energy-driven shape memory piezoelectric nanogenerator (sm-PENG) integrated with a fixation splint to promote osteogenic differentiation for potential bone repair (Fig. 10a). The optimized shape memory structure increased the short-circuit current to 20 μA, more than twice that of the flat structure. After integration with a rectifier bridge, the pulse-DC generated by the sm-PENG effectively elevated pro-osteoblast (MC3T3-E1) proliferation and intracellular Ca^2+^, thus rearranging cell alignment. Meanwhile, the biological effect of pulse-DC stimulation from sm-PENG was consistent with that of a commercial signal generator [197].

Ultrasound vibration can also be selected as a mechanical source to generate piezoelectric stimulation, which shows priority for noninvasiveness, deep tissue penetration, and precise targeting [183, 192, 202]. As an FDA-approved therapeutic tool, ultrasound can effectively compensate for the reduced performance of PENGs caused by subtle and insufficient biomechanical movements. As a highly vascularized tissue, the rich vascular system within bone tissue ensures the survival and activity of osteoblasts and plays a crucial role in maintaining bone formation and repair [163]. To investigate this issue, Chen and coworkers created a US-triggered ZIF-8 decorated hierarchical PVDF foam PENG by decorating ZIF-8 crystals into a PVDF foam sheet (Fig. 10b). Porous microstructures reinforced the flexibility and strain accumulation capability of PVDF/ZIF-8 PENG, achieving sustained release and enrichment of Zn^2+^, which was proved to embody osteogenesis and antibacterial effects [198]. As demonstrated in molecular dynamics simulations, piezoelectric potential triggered Zn^2+^ movement toward and deposition on the surface of PVDF. Subsequent in vitro studies displayed superior properties of PVDF/ZIF-8 PENG on angiogenesis, osteogenesis, and antibacterial effects. Subsequently, the positive influence of PENG on healing calvarium defects was verified, guiding vascularized bone remodeling through ultrasound-induced micro-current and Zn^2+^ enrichment. In the femoral defect model, this PENG reshaped the physiological environment, implementing the synergistic coupling of bone regeneration and angiogenesis. By utilizing RNA-sequencing technology, this study pointed out the up-regulation of oxidative phosphorylation and ATP-coupled cation transmembrane transportation in vascular endothelial cells caused by PVDF/ZIF-8 PENG, significantly promoting BMSCs uptake of Zn^2+^ and activating signaling pathways that regulate angiogenesis and osteogenesis.

Despite the outstanding progress the self-powered PENG device has made in vitro, in vivo applications of PENGs are still in their infancy for bone tissue regeneration, and only a few studies of PENG for bone trauma repair have been reported to date. This is caused by the low voltage output and power density of PENGs themselves. Existing evidence indicates that hybrid NGs integrating TENGs into PENGs, capable of simultaneously collecting multiple forms of energy, have become the most effective method to improve electromechanical conversion efficiency. To date, this kind of tribo-piezoelectric effect-coupling NG has been innovatively developed and successfully used to modulate atrial fibrillation and peripheral nerve restoration [30, 203]. Recently, Wang et al. proposed a rehabilitation exercise-driven symbiotic ES system (BD-ES) composed of a hybrid tribo/piezoelectric nanogenerator (HTP-NG) and a conductive biodegradable hydrogel for the treatment of bone defects (Fig. 10c) [199]. The hybrid structure endowed the HTP-NG with a higher electromechanical conversion ability to capture the biomechanical energy produced by knee joint motions compared to a single TENG or PENG module. The generated electric pulses were then conducted into the defect site for ES therapy. Additionally, inspired by the natural biomineralization process, the PDA-modified black phosphorus (PDA-BP) doped in hydrogel promoted electron transportation and gradually degraded to PO_4_^3−^ for mineral deposition. In vivo evaluation was conducted by injecting the conductive hydrogel into a 3-mm-diameter bone defect region and connecting it to the HTP-NGs fixed in front of a rat’s knee with a Pt wire. Results demonstrated that the femur defect was healed within 6 weeks, and the accelerated bone regeneration was supported by enhanced angiogenesis, skeletal system, and connective tissue development. This study effectively fills the gap in the design and use of hybrid self-powered devices for future personalized healthcare in bone defect treatment.

## Conclusions and Outlook

The integration of electroactive hybrid biomaterials and self-powered systems into the realm of bone therapeutics holds transformative potential, poised to elevate standards of care in bone regeneration and repair. Drawing from numerous studies and innovative strides in this field, it is evident that the convergence of bioengineering, materials science, and nanotechnology can propel us toward unprecedented advancements in medical treatment [204–210]. Electroactive materials, including conductive polymers and piezoelectric substances, offer a unique set of properties that make them ideal for fostering bone growth and healing [211]. These materials excel not only in providing mechanical strength but also in delivering electrical stimulating cues, akin to natural bone’s piezoelectric properties. This intrinsic electrical activity is crucial in modulating cellular functions such as proliferation, differentiation, and matrix production, all pivotal for effective bone regeneration. Hybrid biomaterials, consisting of biodegradable matrices embedded with electroactive components, have demonstrated their capability to enhance the osteogenic potential of scaffolds. The tunability of these composites allows for the customization of mechanical properties, degradation rates, and electroactivity, optimizing the scaffold’s performance to meet specific clinical requirements. For instance, scaffolds incorporating conductive polymers like polypyrrole embedded in biodegradable matrices such as polylactic acid (PLA) have shown significant promise in promoting bone tissue formation. Simultaneously, self-powered systems, particularly those based on nanogenerators such as TENGs and PENGs, harness physiological movements to generate electrical stimuli autonomously. This ability to convert biomechanical energy into electrical signals ensures continuous and localized stimulation at the injury site, thereby overcoming the limitations posed by externally powered devices. Integration of these nanogenerators into bone scaffolds has opened avenues for autonomous and sustained therapeutic interventions, further enhancing the efficacy of bone healing processes. The clinical applications of electroactive hybrid biomaterials and self-powered systems are manifold. These innovations stand to revolutionize treatment strategies for complex bone fractures, large bone defects, and conditions like osteoporosis, where traditional methods fall short. Enhanced vascularization and bone growth facilitated by these advanced materials can significantly reduce healing times and improve patient outcomes, offering a new lease on life to those with debilitating bone conditions.

Looking toward the future, several key areas merit attention for the continued evolution and optimization of electroactive hybrid biomaterials and self-powered systems in bone therapeutics [212–218]. First, the development of more sophisticated and patient-specific designs holds great promise. With the advent of advanced manufacturing techniques such as 3D printing and bioprinting, there is potential to create customized scaffolds that match the anatomical and physiological nuances of individual patients [219–222]. This level of customization will not only enhance the compatibility and performance of implants but also minimize risks of rejection and complications.

Second, a deeper understanding of the molecular and cellular mechanisms underlying the interaction between electrical signals and bone cells is essential. Elucidating these pathways will enable the precise tuning of electrical stimuli to maximize osteogenic responses, fostering more effective regenerative outcomes. Advanced in vitro and in vivo models will play a critical role in this investigative approach, providing insights that can be translated into clinical applications.

Additionally, the scalability and manufacturability of these advanced biomaterials need to be addressed. For widespread clinical adoption, the production of these materials must be cost-effective and scalable while maintaining consistent quality and performance. Collaboration between academia, industry, and regulatory bodies will be crucial to establish standardized protocols and ensure that these innovations can transition from the bench to the bedside.

Moreover, long-term studies on the biointegration and degradation of these materials within the human body are essential to understand their long-term effects and potential risks. Such studies will provide critical data on the biocompatibility, stability, and eventual biodegradation of these materials, ensuring their safety and efficacy for clinical use.

Furthermore, exploring the integration of these materials with other therapeutic modalities such as drug delivery systems and gene therapy could further expand their therapeutic reach. For instance, incorporating drug-loaded nanoparticles within the electroactive scaffold could offer a multifaceted approach to simultaneously stimulate bone growth and deliver localized therapeutics, enhancing the overall treatment efficacy.

Lastly, there is an exciting opportunity to harness advances in wireless technology and remote monitoring systems. Integrating sensors within the scaffold to monitor healing progress and relay data to healthcare providers could pave the way for more proactive and personalized medical care. Intelligent systems capable of adjusting the electrical stimulation based on real-time data could optimize healing processes and offer tailored therapies for individual patients.

In conclusion, the fusion of electroactive hybrid biomaterials and self-powered systems represents a paradigm shift in bone therapeutics. The potential to create dynamic, responsive, and self-sustaining treatment platforms is not just a futuristic aspiration but a tangible possibility on the horizon of medical innovation. As research continues to break new ground and interdisciplinary efforts converge, we stand on the brink of transforming how we approach bone regeneration, repair, and overall patient care, ultimately ushering in a new era of medical excellence and improved quality of life for patients worldwide.
